# Synthetic Lethality of SHP2 and XIAP Suppresses Proliferation and Metastasis in *KRAS*‐mutant Nonsmall Cell Lung Cancer

**DOI:** 10.1002/advs.202411642

**Published:** 2025-02-24

**Authors:** Nai‐jie Fu, Yu‐wen Sheng, Zhe Fan, Zhao Wu, Ling‐yu Li, Rui‐ying Xi, Xiao‐ke Shi, Guo‐lin Zhang, Fei Wang

**Affiliations:** ^1^ Center for Natural Products Research Chengdu Institute of Biology Chinese Academy of Sciences Chengdu 610041 China; ^2^ College of Agriculture Sichuan Agricultural University Chengdu 611130 China; ^3^ University of Chinese Academy of Sciences Beijing 100049 China

**Keywords:** embelin, KRAS, lung cancer, SHP2, XIAP

## Abstract

Kirsten rat sarcoma viral oncogene homolog (*KRAS*) mutations are associated with poor prognosis and poor response to standard therapeutic regimens in patients with nonsmall cell lung cancer (NSCLC). Identification of novel synthetic lethal partners in oncogenic *KRAS* is an alternative therapeutic strategy for *KRAS*‐mutant malignancies. After high‐throughput screening against a preclinical/clinical compound library, embelin, a known X‐linked inhibitor of apoptosis protein (XIAP) inhibitor, specifically inhibits the catalytic activity and phosphorylation of Src homology domain 2 containing tyrosine phosphatase 2 (SHP2) in *KRAS*‐mutant NSCLC cells. Pharmacological inhibition and genetic knockdown of XIAP and SHP2 induce synthetic lethality in *KRAS*‐mutated NSCLC cells and xenograft animal models. Mechanistically, dual inhibition of XIAP and SHP2 by embelin lessens the proliferation and metastasis, activates senescence and endogenous apoptosis, inhibits cancer‐related RAS/mitogen‐activated protein kinase (MAPK), phosphoinositide‐3‐kinase (PI3K)/AKT, Janus kinase/signal transducers and activators of transcription (JAK/STAT), wingless‐related integration site (Wnt), and nuclear factor kappa B (NF‐κB) signaling pathways, and overcomes compensatory feedback in the MAPK signals through the modulation of mitogen‐inducible gene‐6 (MIG‐6) and SPROUTY2 (SPRY2). Collectively, SHP2 and XIAP are potential synthetic lethal partners, and embelin warrants further development as a novel therapeutic option for alleviating *KRAS*‐mutant NSCLC by cotargeting SHP2 and XIAP.

## Graphical abstract

Embelin cotargets SHP2 to disrupt the SHP2/GRB2/SOS complex, inhibiting cancer‐related signaling pathways such as MAPK, (PI3K)/AKT pathway, and XIAP to induce apoptosis signaling pathway, respectively, leading to synthetic lethality in *KRAS*‐mutant NSCLC cells both in vivo and in vitro.

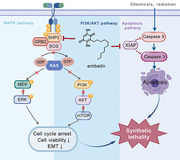

## 1 Introduction

Globally, Kirsten rat sarcoma viral oncogene homolog (KRAS) mutations are the most common potential target molecular subtypes in nonsmall cell lung cancer (NSCLC), with an incidence rate of 20%–25% in Western countries and approximately 10%–15% of cases in Asia.^[^ ^1^ ^]^ KRAS functions as a critical cellular signaling switch, alternating between its inactive GDP‐bound form (GDP‐KRAS) and its active GTP‐bound form (GTP‐KRAS). This switch transmits extracellular signals from the cell surface to the nucleus by interacting with different downstream effectors.^[^ ^2^ ^]^ *KRAS* mutations result in impaired GTP hydrolase activity, which promotes the continued generation of activated GTP‐KRAS and the continued activation of downstream signaling pathways, allowing substrates, including a variety of kinases and transcription factors, to be phosphorylated by extracellular signal‐regulated kinases (ERK).^[^ ^3^ ^]^ Numerous strategies have been developed, including direct targeting of KRAS or inhibition of modifying enzymes required for KRAS membrane binding.^[^ ^2^, ^4^ ^]^ To date, only *KRAS* ^G12C^ and *KRAS* ^G12D^ inhibitors have been used in clinical studies.^[^ ^5^, ^6^ ^]^ However, some treated cancer cells acquire resistance by generating novel *KRAS* ^G12C^ mutants that evade compound inhibition.^[^ ^7^ ^]^ In addition, most inhibitors cannot enter the clinical phase owing to the limitations of low selectivity and activity, metabolic instability, drug resistance, and obvious toxic side effects.^[^ ^7^, ^8^ ^]^ Moreover, covalent inhibitors require *KRAS* ^G12C^ mutants to be in an inactive GDP‐KRAS state, which limits their clinical application.^[^ ^9^ ^]^ Therefore, identifying other options for targeting *KRAS* mutations is essential.

Several potential targets for KRAS synthetic lethality have been identified, including cyclin‐dependent kinase 1 (CDK1) and Src homologous region 2 containing protein tyrosine phosphatase 2 (SHP2).^[^ ^10^, ^11^ ^]^ SHP2, encoded by the *PTPN11* gene, is an evolutionarily conserved non‐receptor‐type protein tyrosine phosphatase (PTP) consisting of two SH2 structural domains (N‐SH2 and C‐SH2) and a PTP catalytic domain.^[^ ^12^ ^]^ SHP2 is currently the only confirmed proto‐oncoprotein in the PTP family and is a core component of the signaling protein complex downstream of activated receptor tyrosine kinases (RTKs), which promote KRAS activation through the guanine exchange factor SOS1.^[^ ^11^ ^]^ In addition, SHP2 can dephosphorylate the negative regulatory factor Sprouty, reducing its inhibitory effect on the Grb2/SOS complex, thereby activating the RAS/mitogen‐activated protein kinase (MAPK) pathway.^[^ ^13^, ^14^ ^]^ Although tyrosine kinase inhibitors (TKIs) initially suppress the MAPK signaling pathway, MAP kinase (MEK) and ERK are reactivated concomitantly with enhanced SHP2 phosphorylation and activity, which is considered the major cause of fewer clinical benefits in treating *KRAS*‐mutant tumors by the combination of MEK inhibitors and TKIs.^[^ ^15^, ^16^ ^]^ Recent studies have reported the discovery of allosteric inhibitors of SHP2, such as SHP099 and RMC‐4630, and showed their efficacy in inhibiting TKI‐induced ERK‐negative feedback in *KRAS*‐mutant tumors.^[^ ^16^, ^17^ ^]^ In addition, SHP2 interacts with and regulates the downstream receptors of the immune receptor tyrosine inhibitory motif or the activation domain of cytotoxic T lymphocyte‐associated antigen‐4 (CTLA‐4), programmed death receptor 1 (PD‐1), CD272, T cell Ig, and immune receptor tyrosine inhibitory motif domain (TIGIT), playing an important role in T cell‐mediated immune responses.^[^ ^18^, ^19^ ^]^ Therefore, targeting SHP2 or combination therapy is highly anticipated in the clinic as a small‐molecule tumor immunotherapy drug. However, the mono‐therapeutic efficacy of TNO155, a derivative of SHP099, is not promising in solid tumors, and Phase Ib clinical data on the *KRAS* ^G12C^ inhibitor Lumakras in combination with RMC4630 showed that combination therapy was not superior to Lumakras alone.^[^ ^20^, ^21^ ^]^ Although clinical investigations of TNO155 with the inhibitors of *KRAS* ^G12C^, EGFR, BRAF, MEK, ERK, and PD‐1 are ongoing for multiple solid cancers, novel synthetic lethal patterns of SHP2 must be discovered to further enhance its clinical efficacy of SHP2 inhibitors.

XIAP is a member of the inhibitor of apoptosis protein (IAPs) family and is known for its important role in modulating cell death by inhibiting the initiator caspase 9 and effector caspase 3/7.^[^ ^22^, ^23^ ^]^ XIAP is overexpressed in most tumor cell lines and is closely associated with tumor progression, recurrence, prognosis, and resistance to chemotherapy.^[^ ^24^, ^25^ ^]^ Therefore, XIAP has received considerable attention from the pharmaceutical industry as a promising therapeutic target for cancer, and multiple agents have progressed to clinical trials.^[^ ^24^ ^]^ However, in a transient overexpression system, XIAP could only inhibit apoptosis at levels exceeding its physiological concentration.^[^ ^26^ ^]^ Prolonged expression at comparable concentrations in tumor cells results in little or no resistance to chemotherapeutic agents such as doxorubicin, vinblastine, and vincristine, and severe resistance is produced only in the presence of XIAP overexpression and stable knockdown of XIAP antagonists, including SMAC and XAF1.^[^ ^27^, ^28^, ^29^ ^]^ In addition, the precise regulatory balance between XIAP and its antagonists was found to be crucial for determining the clinical outcomes of patients with cancer in an analysis of 187 cases of gastric adenocarcinoma. Hence, the function of XIAP depends on itself as well as on its accompanying regulatory factors. To predict the antitumor effect of chemical drugs, the expression levels and functional status of XIAP modulators need to be considered, but the underlying mechanism is currently unknown.

Here, we screened a preclinical/clinical compound library consisting of 3471 bioactive compounds and identified embelin, a potent nonpeptide cell‐permeable inhibitor of XIAP, specifically targeting SHP2, establishing a connection between these two cancer‐related proteins for the first time. To verify the function of SHP2 and XIAP in LUAC, we analyzed The Cancer Genome Atlas (TCGA) database and found that both XIAP and SHP2 were highly correlated with KRAS, and both pharmacological inhibition and genetic knockdown of SHP2 and XIAP resulted in marked cell growth inhibition in *KRAS*‐mutant NSCLC in a synergistic manner. As a dual‐target inhibitor, embelin decreases proliferation, induces cellular senescence, reverses epithelial mesenchymal transformation (EMT), overcomes negative feedback from the MAPK signaling pathway, induces apoptosis, and thus reduces the malignant phenotype of *KRAS*‐mutant NSCLC, both in vivo and in vitro. This study provides a compound backbone for the development of novel SHP2 inhibitors and an experimental basis for preclinical studies based on SHP2 and XIAP dual‐target inhibition, which serve as clinically actionable targets for the treatment of *KRAS*‐mutant NSCLC.

## 2 Results

### 2.1 Embelin Inhibits *KRAS* Mutant Tumor Development

**Figure 1:**
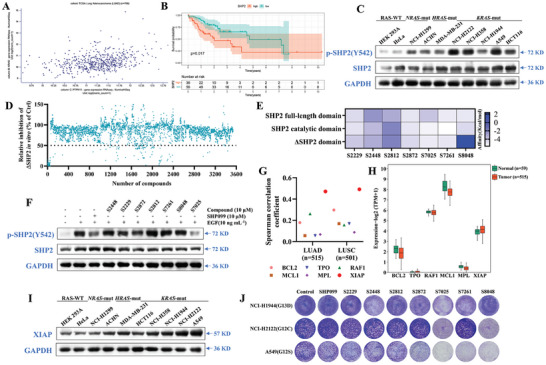
Embelin inhibits *KRAS*‐mutant tumor development. A) Correlation detection between mRNA expression of *PTPN11* and *KRAS* in patients with LUAD (*n* = 706) using IlluminaHiSeq analysis with data derived from TCGA database. B) Correlation and number at risk of SHP2 expression with the overall survival probability of *KRAS* mutant lung cancer patients (*n* = 92) in the TCGA database. C) Protein expression and phosphorylation levels of SHP2 in *RAS*‐WT, *NRAS*, *HRAS*, and *KRAS*‐mutant cells. D) Effect of 3471 bioactive compounds at 10 µM against SHP2, with Na_3_VO_4_ serving as a control. Z’ factor values for 69 plates screened, Z’ = 0.81, S/N = 40.8. E) Binding energy between candidate drugs and SHP2 full‐length domain, catalytic region, and ΔSHP2 domain. F) HeLa cells were pretreated with DMSO, SHP099, or various compounds at 20 µM for 2 h before stimulation with EGF (10 ng mL^−1^) for an additional 2 h. Cell lysates were subjected to immunoblotting with SHP2, p‐SHP2, and GAPDH antibodies. Data are from three independent experiments are presented. G) Spearman's correlation coefficient between drug‐related targets and *KRAS* mRNA in different types of tumors through the TCGA database. H) *BCL2*, *TPO*, *RAF1*, *MCL1*, *MPL*, and *XIAP* mRNA expression levels in LUAD tumors and adjacent tissues through the TCGA database. I) XIAP protein expression in *RAS*‐WT, *NRAS*‐mutant, *HRAS*‐mutant, and *KRAS*‐mutant cells. J) Effect of candidate drugs (10 µM) on colony formation in A549, NCI‐H2122, and NCI‐H1944 cells.

To detect the correlation between *KRAS* and *PTPN11*, we screened the gene co‐expression of lung adenocarcinoma (LUAD) patients (*n* = 706) in TCGA database. The results indicated a strong positive correlation between *PTPN11* and *KRAS* with a Pearson's correlation coefficient R = 0.3361 (*p* = 1.121×10^−16^), and Spearman's rank coefficient Rho = 0.3178 (*p* = 5.502 × 10^−15^) (**Figure** **1**A). Moreover, we obtained KRAS‐SHP2 PPIs through the STRING database and found that SHP2 interacted with KRAS directly as well as indirectly through RAF1, PDCD1, CTLA4, BRAF, EGFR, GAB1, IGF1R, PIK3CA, IRS1, and NTRK2 (Figure S1A, Supporting Information), suggesting that targeting SHP2 could affect the course of KRAS‐associated diseases. Furthermore, we found that patients with high *PTPN11* expression had a lower overall survival (OS) than those with low expression (Figure S1B, Supporting Information). To assess the prognostic potential of SHP2, we conducted a subgroup analysis of the patient cohort according to their KRAS mutation status. A significant association between low SHP2 expression and improved prognosis was observed in both *KRAS* mutant NSCLC and LUAD subgroups. Specifically, patients with low SHP2 expression in the *KRAS* mutant NSCLC subgroup demonstrated a better overall survival rate, with a *p*‐value of 0.017 (Figure 1B). Similarly, in the *KRAS* mutant LUAD subgroup, low SHP2 expression was associated with a favorable prognosis, with a *p*‐value of 0.015 (Figure S1C, Supporting Information). In addition, the clinical progression stage of the patients was significantly positively correlated with high expression of *PTPN11* (Figure S1E, Supporting Information). In an effort to further elucidate the expression patterns of SHP2 in a clinical context, we conducted protein expression analysis in *KRAS* wild‐type and mutant tissues within LUAD and lung squamous cell carcinoma (LUSC) from the TCGA database. Our analysis showed that the expression of SHP2 in *KRAS* wild‐type LUAD tissues (*n* = 269) was significantly lower compared to *KRAS* mutant group (*n* = 85), with *p*‐values of 0.0423 (Figure S1D, Supporting information). Consistent with the above results, we found that SHP2 was expressed at a lower level in normal human embryonic kidney HEK293A cells and phosphorylated to a lower degree in *RAS* wild‐type HEK293A and HeLa cells; the phosphorylation level was significantly elevated in *RAS*‐mutant cells, especially in the *KRAS*‐mutant tumor cell lines (Figure 1C). However, our analysis revealed no statistical differences in overall survival for *KRAS* mutant LUSC, due to the limited number of samples available for this particular subgroup (Figure S2A, Supporting Information). In contrast, low SHP2 expression did not show a good prognosis in *KRAS* wild‐type lung cancer, LUAD, and LUSC patients (Figure S2B–D, Supporting Information). In summary, our analysis demonstrates that SHP2 expression emerges as a promising prognostic biomarker specifically in *KRAS* mutant lung cancer subgroups.

Therefore, we performed activity assays by establishing an SHP2 activity‐screening model against a preclinical/clinical compound library consisting of 3471 bioactive compounds. The screening identified seven compounds with specificities for SHP2 and other PTPs (Figure 1D; Figure S1F, Supporting Information, and **Table** **1**). After molecular docking with the full‐length region, the catalytic region, and the N‐SH2‐free region of SHP2 (ΔSHP2), we found that S2229, S7025, and S2872 were more tightly bound to SHP2 (Figure 1E). SHP2 inhibitors identified by in vitro screening often fail to inhibit SHP2 phosphorylation because of permeability issues, whereas the level of phosphorylation is closely related to the degree of tumor malignancy. Therefore, we examined the effect of the candidate compounds on the level of SHP2 phosphorylation. Both S2872 and S7025 significantly inhibited SHP2 phosphorylation (Figure 1F). In addition, we obtained the correlation between drug‐related targets and *KRAS* by checking the TCGA database and revealed that *XIAP* was positively correlated with *KRAS* gene expression in LUAD and LUSC tumors with the highest relevance. *XIAP* expression was significantly higher in LUAD tumor tissues than in normal tissues (*p* = 0.00015), whereas there was no difference in LUSC (Figure 1G H; Figure S1G, Supporting Information). Meanwhile, Our analysis revealed a significant increase in the mRNA expression of the SHP2 and XIAP gene in *KRAS* mutant cell lines compared to their wild‐type group, with *p* = 0.0075 and *p* = 0.0392, respectively, from the TCGA database (Figure S1H, Supporting Information). Similarly, the protein expression of SHP2 and XIAP in *KRAS* wild‐type cells was significantly lower compared to *KRAS* mutant cells, with *p*‐values of 0.0430 and 0.0381, respectively, from the Human Protein Atlas (HPA) database (Figure S1I, Supporting Information). In agreement with the above results, we found that XIAP expression was lower in *RAS* wild‐type HEK293A and HeLa cells and was significantly higher in *RAS*‐mutant cells, particularly in *KRAS*‐mutant tumor cells (Figure 1I), suggesting that targeting XIAP could also affect the course of *KRAS*‐related diseases. In contrast, our analysis revealed that low expression of XIAP does not correlate with a favorable prognosis in *KRAS* mutant lung cancer, including both LUAD and LUSC (Figure S3A–F, Supporting Information), indicating that the expression level of XIAP might not be used as an independent prognostic marker in these subsets of *KRAS* mutant lung cancer.

**Table 1:** Candidate compound‐related targets and inhibitory effects on PTPs.

Number	Name	Target	IC_50_ of PTPs [µM]					
			HePTP	TCPTP	VHR	PTP1B	ΔSHP1	ΔSHP2
S2229	Eltrombopag^a^	TpoR	201.9	>200	241.8	145.3	>200	21.69
S2448	β‐Guttiferin^b^	Caspases	>200	293.7	123.01	30.33	>200	11.76
S2812	AT101	Mcl‐1	134.83	197.4	141.24	77.62	196.7	39.53
S2872	GW5074	c‐Raf	472	321.6	131.39	109.5	245.7	33.81
S7025	Embelin	XIAP	>200	>200	>200	>200	>200	10.49
S7261	ARQ‐501^c^	TOPO	>200	>200	>200	>200	>200	34.23
S8048	Venetoclax	Bcl‐2	19.97	9.43	36.86	16.61	81.3	2.065

^a)^ Eltrombopag is short for Eltrombopag Olamine;

^b)^ β‐Guttiferin is an alternative name for Gambogic acid;

^c)^ ARQ‐501 is another name for β‐Lapachone.

**Figure 2:**
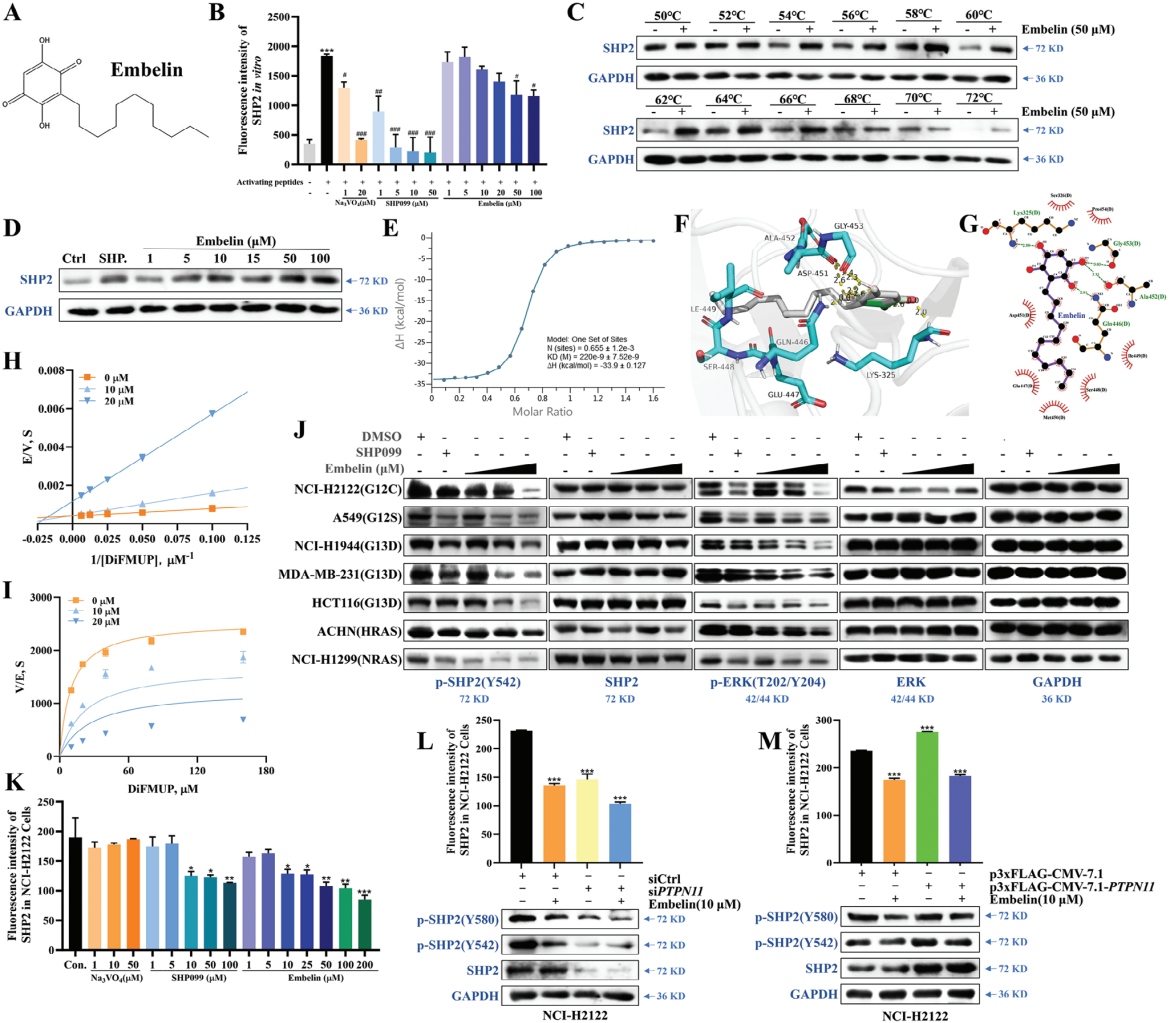
Embelin as a novel SHP2 catalytic inhibitor. A) Chemical structure of embelin. B) Inhibitory effect of embelin, SHP099, and Na_3_VO_4_ on PTPase activity of full‐length SHP2 domain in vitro. ^#^ *p *< 0.05 compared to DMSO group; ^##^ *p* < 0.01 compared to DMSO group, and ^###^ *p *< 0.001 compared to DMSO group. ^***^ *p *< 0.001 compared to the negative control group (unpaired two‐tailed Student's *t*‐test). C, D) Binding of embelin with SHP2 at different temperatures (C) and different concentrations at 58 °C (D) was assessed using CETSA, with SHP099 (20 µM) as control. E) ITC titration results of embelin and His‐ΔSHP2, reflected in the fitting diagram of the 1:1 binding mode. F, G) embelin was docked to ΔSHP2 domain (F). Important binding residues and amino acids are shown (G). H) Lineweaver‐Burk plot of SHP2 inhibition by embelin. Enzyme kinetics of SHP2 with varying concentrations of DiFMUP and embelin. I) Michaelis‐Menten model of SHP2 inhibition by embelin. Enzyme kinetics of SHP2 with varying concentrations of substrate DiFMUP and embelin (0, 10, and 20 µM). J) Effects of embelin and SHP099 on SHP2, ERK expression, and phosphorylation in *HRAS*, *NRAS*, and *KRAS* mutant cells at normal serum concentrations. K) Inhibitory effect of embelin, SHP099, and Na_3_VO_4_ on PTPase activity of SHP2 in NCI‐H2122 cells. L, M) si*PTPN11* L) or p3×Flag‐CMV‐7.1‐*PTPN11* M) was transfected into NCI‐H2122 cells for 24 h, then embelin was added for 24 h. Expression and phosphorylation of SHP2 in cell lysates were detected using western blotting and SHP2 catalytic activity was analyzed using co‐IP and fluorescence reading. ^***^ *p *< 0.001 compared to siCtrl group or p3×Flag‐CMV‐7.1 group (unpaired two‐tailed Student's *t*‐test). All experiments were conducted with three independent replicates.

We also examined the effects of the candidate compounds on *KRAS*‐mutant cells. The results showed that S2229 and S7025 inhibited the proliferation of different *KRAS*‐mutation cells at different concentrations, among which S7025 was the most effective (Figure 1J; Figure S1J–L, Supporting Information), and S2229, S2448, and S7261 induced senescence of NCI‐H2122 cells, among which S7025 was the most effective (Figure S1M, Supporting Information). In addition, we found that different XIAP inhibitors (GDC‐0152, Birinapant and BV‐6) and a second mitochondrial activator of caspase (SMAC) analog (LCL‐161) induced cellular senescence (Figure S1N, Supporting Information). Based on these results, we identified that S7025, known as embelin, ameliorates *KRAS*‐mutant NSCLC (**Figure** **2**A).

### 2.2 Embelin is a Novel SHP2 Catalytic Inhibitor

The chemical structure of embelin is shown in Figure 2A. The molecular docking results indicated that embelin showed almost no binding to CDC25A, CDC25B, DUSP6, LMPTP, PRL‐1, PRL‐3, PTP1B, PTPN22, RPTPδ, SHP1, STEP, and VEPTP (Figure S4A,B, Supporting Information). In addition, embelin had no effect on SHP2 full‐length activity allosterically activated by the bis‐phosphorylated peptide from Gab1, which dissociated the N‐SH2 region from the C‐SH2 terminus to expose the PTP‐active region, suggesting that embelin is a potential novel allosteric inhibitor of SHP2 catalytic activity (Figure 2B). The PTP region of the PTP family members is highly conserved. SHP1 and SHP2 are non‐receptor‐like PTPs with 55% sequence homology and similar 3D structures and catalytic activation mechanisms. embelin had little inhibitory effect on other PTPs, including SHP1 (Table 1).

We examined the ability of embelin to bind to SHP2 using CETSA and ITC. CETSA revealed that embelin increased the stability of SHP2 protein in a concentration‐dependent manner (Figure 2C,D). ITC showed that embelin bound to SHP2 and ΔSHP2, and the K_D_ value was 22.9 and 220 nM, respectively (Figure 2E; Figure S4E,G, Supporting Information). To further evaluate the binding sites of embelin and SHP2, we performed molecular docking using the published SHP2 structure (PDBID: 5EHR) and ΔSHP2 structure (PDBID: 7PPL), and results showed that embelin formed hydrogen bonds with Lys^325^, Gly^453^, Ala^452^, and Gln^446^ residues of ΔSHP2 protein, hydrophobic interactions with Asp^451^, Ile^449^, Ser^448^ of ΔSHP2 protein; a hydrogen bond with His^114^ in the helical region of the SHP2 protein, and hydrophobic interaction with Glu^249^, Glu^250^, Thr^218^, His^116^, and Arg^111^, respectively, which suggested that embelin is involved in binding to the SHP2 catalytic structural domain and thus inhibiting SHP2 catalytic activity (Figure 2F,G; Figure S4C,D, Supporting Information). Furthermore, a ΔSHP2^K325A^ mutant that contains the mutation in the Lys^325^ residue to alanine was constructed to examine its interaction with embelin. Interestingly, compared with the wild‐type ΔSHP2, the enzymatic activity of ΔSHP2^K325A^ significantly decreased (*p *< 0.0001, Figure S4H, Supporting Information), and this mutation notably diminished the inhibitory effect of embelin on SHP2 activity. These results suggest that Lys^325^ plays an indispensable role in the enzymatic function of SHP2. The specific interactions observed with ΔSHP2^K325A^ and ΔSHP2 with embelin were subsequently confirmed by ITC analysis using recombinant expressed proteins. The results revealed that compared with the wild‐type ΔSHP2, the exothermic reaction between embelin and ΔSHP2^K325A^ was significantly reduced, and the K_D_ value increased from 220 to 346 nM (Figure S4E–G, Supporting information). These results indicated that Lys^325^, which is located in the catalytic domain, participates in the interaction with embelin. Our protein and compound dialysis results indicated that embelin binds to SHP2 in a noncovalent manner (Figure S4I, Supporting Information). In addition, Km of ΔSHP2 protein was unchanged, whereas Vm was decreased after the addition of embelin, indicating that embelin was a noncompetitive inhibitor of SHP2 (Figure 2H,I).

To investigate whether embelin affects SHP2 at the cellular level, we examined the effects of embelin on SHP2 activity and phosphorylation levels in *KRAS*‐mutant and wild‐type cells. The results showed that embelin inhibited SHP2 phosphorylation in *KRAS* ^G12C^, *KRAS* ^G12S^, *KRAS* ^G13D^, *HRAS*, and *NRAS*‐mutant cells, whereas downstream ERK phosphorylation was also significantly reduced (Figure 2J). In addition, embelin significantly inhibited intracellular SHP2 activity (Figure 2K). Next, we validated the cellular targets of embelin by overexpressing and knocking down SHP2. The results showed compared with the untransfected cells, embelin inhibited the activity and phosphorylation of SHP2 in SHP2 overexpressed cells, and knockdown of SHP2 attenuated the inhibitory effect of embelin on SHP2 phosphorylation and activity (Figure 2L,M; Figure S4J,K, Supporting Information). These results indicate that embelin affects the conformation of SHP2 by binding to existing SHP2 within the cell, thereby inhibiting its phosphorylation level and activity.

Furthermore, we found that SHP2 inhibitors (NSC‐87877, SHP099, and Na_3_VO_4_) did not affect XIAP protein stability other than embelin and GDC‐0152 by CETSA or XIAP expression in NCI‐2122 and A549 cells (Figure S5A,B, Supporting Information). We also tested the effects of other XIAP inhibitors (GDC‐0152, Birinapant, and BV‐6) and a SMAC analog (LCL‐161) on SHP2 expression, phosphorylation, and activity. These structurally different XIAP inhibitors did not affect SHP2 protein stability by CETSA, nor did they affect SHP2 phosphorylation or activity (Figure S5C–F, Supporting Information). We also examined the effects of knocking down SHP2 and XIAP individually and simultaneously on their expression and phosphorylation, and discovered that SHP2 knockdown did not affect XIAP expression and that knockdown of XIAP had no effect on SHP2 expression and phosphorylation (Figure S5G–I, Supporting Information). By exploring TCGA and STRING databases, we found that XIAP had no direct effect on SHP2 and correlated poorly with the Pearson correlation coefficient R = 0.1705 and Spearman's correlation coefficient Rho = 0.1632 (Figure S5J,K, Supporting Information). In summary, the inhibitory effect of embelin on SHP2 is not mediated by XIAP, and SHP2 and XIAP are independent and important intracellular targets of embelin.

Due to the poor membrane permeability of SHP2 catalytic inhibitors,^[^ ^18^, ^30^ ^]^ we examined the effect of embelin on SHP2 expression and phosphorylation after removing the compound from the culture media of A549 and NCI‐H2122 cells. The results showed that continuous treatment with embelin for 24 h significantly inhibited the phosphorylation of SHP2 and decreased XIAP expression, and removal of embelin after 6 h of treatment partially reversed these changes, suggesting that embelin has strong membrane permeability (Figure S5L, Supporting Information).

### 2.3 Embelin Inhibits Proliferation and Metastasis in *KRAS*‐Mutant NSCLC Cells both In Vitro and In Vivo

**Figure 3:**
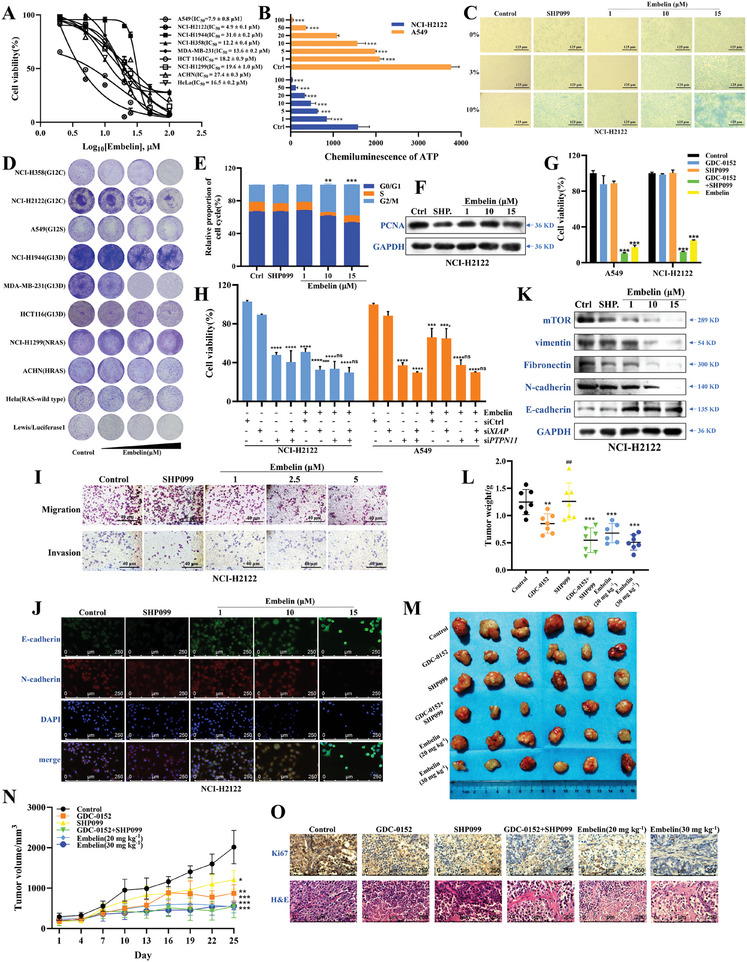
Embelin inhibits proliferation and metastasis in *KRAS*‐mutant NSCLC cells both in vitro and in vivo. A) The effect of embelin on cell viability across a mini‐panel of *KRAS*‐mutant and ‐wild‐type cell lines after 24 h was measured using the CCK‐8. Bars, ± SEM. The curves were plotted using a variable slope (four‐parameter) nonlinear fit. B) Cells were treated with embelin for 48 h in 3D culture cell mode, and the intracellular ATP chemiluminescence was detected. ^***^ *p *< 0.001 compared to *the* control group (unpaired two‐tailed Student's *t*‐test). C) Effects of embelin and SHP099 inducing senescence shown using β‐galactosidase staining in human NCI‐H2122 cells (×40). D) Effects of colony formation of embelin in *KRAS* wild‐type and mutant cells. E) Statistical graph of cycle ratio of NCI‐H2122 cells after treating with various concentrations of embelin. ^**^ *p *< 0.01 and ^***^ *p *< 0.001 compared to *the* control group (one‐way ANOVA). F) Western blotting of PCNA protein expression after treating with various concentrations of embelin. G) Effects of SHP099 and GDC‐0152 separately or in combination with SHP099 and embelin for 24 h on the cell viability determined using the CCK‐8. ^***^ *p *< 0.001 compared to *the* control group (unpaired two‐tailed Student's *t*‐test). H) Cells were instantaneously transfected with si*PTPN11* and si*XIAP* and with both for 24 h, and embelin was added for 24 h. Cell proliferation was detected using the CCK8. ^***^ *p* < 0.001 and ^****^ *p* < 0.0001 compared to siCtrl group, and ^ns^ *p* > 0.05 compared to relative groups (unpaired two‐tailed Student's *t*‐test). I) NCI‐H2122 cells were inoculated into the upper lumen of a Transwell cell (the upper layer of the cell was coated with Matrigel for invasion analysis) and treated with embelin and SHP099 (10 µM) for 24 h. Images of cell migration (above) and invasion (below) were captured using a microscope. J) After treatment with embelin and SHP099 (10 µM) for 24 h, expression of EMT‐labeled protein in NCI‐H2122 cells was detected using IF. K) Western blotting was performed to assess protein expression of EMT markers in NCI‐H2122 cells, including E‐cadherin, N‐cadherin, vimentin, and fibronectin, and to verify mTOR protein expression. The above experiments were conducted with three independent replicates. L, M) Tumors were excised from mice at the termination of the experiment. Representative images of mice with xenograft tumors were captured using a camera (M) and weights were measured (L) (*n *= 6, each group). ^**^ *p* < 0.01 and ^***^ *p* < 0.001 compared to control group, and ^##^ *p* < 0.01 compared to GDC‐0152 + SHP099 groups (one‐way ANOVA). N) Tumor volume of BALb/c nude mice with NCI‐H2122 subcutaneous transplantation tumors were measured every 3 days. GraphPad was used to conduct statistical analysis of data (*n *= 6, each group). ^*^ *p* < 0.05, ^**^ *p* < 0.01, and ^***^ *p* < 0.001 compared to the control group (unpaired two‐tailed Student's *t*‐test). O) IHC analyses of Ki67 expression and H&E staining of tumor tissue (×200). (*n *= 6, each group).

The antitumor activity of embelin was examined in *KRAS*‐mutant and wild‐type cells. The CCK‐8 results showed that embelin inhibited the proliferation of various *KRAS*‐mutant and wild‐type cells, whereas NCI‐H2122 and A549 cells were more sensitive to embelin and thus were selected as models for subsequent mechanistic studies (**Figure** **3**A; Figure S6A, Supporting Information). The TCGA database showed *XIAP* and *PTPN11* were positively correlated with the expression of *KRAS* and *NRAS* genes, and negatively correlated with *HRAS* in different types of tumors, with Rho = 0.47 (*p* = 8.46 × 10^−30^) between *XIAP* and *KRAS*, and Rho = 0.58 (*p* = 2.47 × 10^−47^) between *PTPN11* and *KRAS* in LUAD, indicating that embelin has a stronger effect on *KRAS* mutant tumors by targeting XIAP with SHP2 (Figure S6F,G, Supporting Information). To further verify the specificity of embelin on *KRAS* mutant tumor cells, a diverse range of cancer cell lines that contain different *RAS* mutations and originate from various tumor types were used. The data reveals variability in the sensitivity of cancer cell lines to embelin, correlated with their *RAS* mutation status (Table S7, Supplementary Table1). Notably, cell lines with *KRAS* mutations demonstrate increased sensitivity to embelin, with an average IC_50_ value of 16.93 µM, compared with RAS wild‐type cell lines with an average IC_50_ value of 30.82 µM, average IC_50_ values of 44.00 µM for *HRAS* mutants and 19.6 µM for the *NRAS* mutant strain, suggesting that embelin may exert a preferential cytotoxic effect on cell lines with *KRAS* mutations (Figure S6I,J; Table S7, Supplementary Table1). Considering that *KRAS* mutant NSCLC may develop primary resistance to EGFR tyrosine kinase inhibitors (EGFR‐TKI) and *EGFR^T790M^* mutation causes over 50% of acquired resistance to EGFR‐TKIs, A549 cells with *KRAS* mutation, and NCI‐H1975 cells with *EGFR^L858R^* and *EGFR^T790M^* double mutation were used. The results showed that the IC_50_ values for erlotinib and gefitinib in A549 cells were >100 µM and 26.14 ± 0.81 µM, respectively, while the IC_50_ values for these inhibitors in NCI‐H1975 cells were >100 µM and 31.68 ± 4.21 µM, respectively. However, the IC_50_ values for embelin in A549 and NCI‐H1975 cells were 7.9 and 4.948 µM, respectively, suggesting that embelin remains effective against primary and acquired EGFR resistant cells (Figure S6H,I,K‐N, Supporting information). The intracellular ATP assay revealed that embelin significantly inhibited ATP release in NCI‐H2122 and A549 cells and was more effective in 3D cell culture (Figure 3B; Figure S6B, Supporting Information). β‐Galactosidase staining showed that embelin induced senescence in A549, NCI‐H2122, and NCI‐H1944 cells (Figure 3C; Figure S6D,E, Supporting Information). Colony formation assay showed that embelin had long‐term cytotoxic effects on both *RAS* wild‐type and mutant cells (Figure 3D). The cell cycle assay also revealed that embelin blocked NCI‐H2122 cells in the G2/M phase (Figure 3E; Figure S6C, Supporting Information). We also observed that the expression of PCNA, an important marker of cell proliferation and DNA synthesis, was reduced in NCI‐H2122 cells after incubation with embelin (Figure 3F).

To investigate the mechanism by which embelin affects cell survival, we knocked down XIAP and SHP2 expression or added XIAP and SHP2 inhibitors to cancer cells. The results showed that the SHP2 inhibitors SHP099, NSC‐87877, Na_3_VO_4_, Smac analog birinapant, XIAP inhibitor GDC‐0152, and LCL‐161 had almost no effect on cell proliferation in the monolayer cell culture mode, except for the XIAP inhibitor BV‐6, which partially inhibited cell proliferation (Figure S7A–I, Supporting Information). In addition, the combination of GDC0152 and SHP099 significantly inhibited the proliferation of A549 and NCI‐H2122 cells (Figure 3G; Figure S7K, Supporting Information). In 3D cell culture, SHP099 and GDC‐0152 partially inhibited cell proliferation, which was significantly inhibited by the combination (Figure S7J, Supporting Information), suggesting that simultaneous targeting of XIAP and SHP2 has a synergistic lethal or synthetic lethal effect. In addition, cell proliferation was partially attenuated after the respective knockdown of XIAP and SHP2 expression in the monolayer and 3D cell culture models, and was significantly reduced after the combined application (Figure S7L–N, Supporting Information). When embelin was added again, cell proliferation was not inhibited further after the knockdown of SHP2 or combined with si*XIAP*, while cell proliferation was additionally suppressed after XIAP expression was downregulated (Figure 3H). These results suggest that XIAP and SHP2 mediate the anticancer effects of embelin.

To explore the effect of embelin on the mobility of *KRAS*‐mutant NSCLC cells, we examined the effect of embelin on EMT in cells using an EMT functional assay at the molecular level. The results of cell scratch and cell migration assays showed that SHP099 had a poor effect on cell mobility, whereas embelin could potently inhibit the migration capabilities of NCI‐H2122 and A549 cells (Figure 3I; Figure S8A–C, Supporting Information). The cell invasion assay showed that both 1 and 2.5 µM of embelin and SHP099 significantly inhibited the invasive ability of A549 and NCI‐H2122 cells (Figure 3I; Figure S8C, Supporting Information). The results showed that the migratory and invasive abilities of the cells were not affected after the knockdown of XIAP and SHP2 alone and were significantly reduced after combination treatment (Figure S8F,G, Supporting Information). IF and western blotting experiments showed that embelin promoted the expression of the epithelial marker cell protein E‐cadherin; and decreased the expression of the mesenchymal cell‐specific proteins vimentin, Fibronectin, and N‐cadherin (Figure 3J,K; Figure S8D,E, Supporting Information); and reduced mTOR protein expression in NCI‐H2122 cells (Figure 3K). Moreover, the knockdown of XIAP and SHP2 alone elevated E‐cadherin expression and decreased vimentin and fibronectin expression in the cells, and this combination enhanced these changes (Figure S8H, Supporting Information). These results suggested that targeting XIAP and SHP2 could reduce cell mobility by reversing EMT.

To investigate the antitumor effects of embelin in vivo, we established a BALB/c‐nu mouse model of lung cancer by subcutaneous transplantation of NCI‐H2122 tumor cells. The body weight of the mice was monitored every 3 days to assess the safety of the drug. SHP099 and GDC‐0152 administered alone and in combination had little or no effect on the body weight of the mice, which was similar to the effect of embelin (Figure S9B, Supporting Information). H&E staining and organ weight assay showed that GDC‐0152 and SHP099 alone and in combination with embelin had no significant effect on the weights of hearts, livers, lungs, and kidneys, and the structure of hearts and kidneys in mice (Figure S9A,C,D,F,G, Supporting Information). In addition, we found that intraperitoneal injection of embelin (20 and 30 mg kg^−1^) significantly inhibited the tumor growth rate in vivo and was more effective than GDC‐0152 (20 mg kg^−1^) and SHP099 (20 mg kg^−1^) alone. Tumor proliferation after administration of a combination of GDC‐0152 and SHP099 was also significantly inhibited (Figure 3L–N). H&E and Ki67 staining also showed that embelin and the combination of GDC‐0152 and SHP099 inhibited tumor proliferation (Figure 3O).

### 2.4 Embelin Induces Apoptosis in Human *KRAS*‐Mutant NSCLC Cells

**Figure 4:**
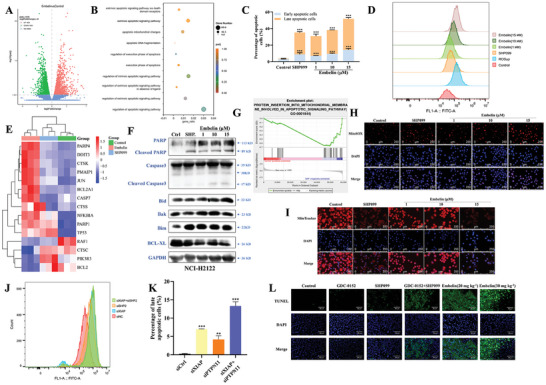
Embelin induces apoptosis in human *KRAS*‐mutant NSCLC cells. A) Volcano map of DEGs (*p*adj value < 0.05, |log2foldchange| > 0). B) Scatter map of GO enrichment analysis of apoptosis‐related biological process obtained by cluster analysis of differential genes between control and embelin group. C) NCI‐H2122 cells were treated with embelin and SHP099 for 24 h and apoptotic cells were detected through Annexin‐V‐FITC/PI double staining. Percentages of apoptotic NCI‐H2122 cells are shown. ^***^ *p *< 0.001 compared to the control group (unpaired two‐tailed Student's *t*‐test). The experiments were conducted with three independent replicates. D) NCI‐H2122 cells were treated with embelin and SHP099 (10 µM) for 24 h. The effect of ROS release and fluorescence intensity of NCI‐H2122 cells were detected. Rosup was used as a positive control. The experiments were conducted with three independent replicates. E) Heatmap hierarchical clustering displayed DEGs in the apoptosis biological processes between the control, SHP099, and embelin groups through RNAseq with *p*adj < 0.05. F) NCI‐H2122 cells were treated with embelin and SHP099 (10 µM) for 24 h and the expressions of apoptosis‐related proteins were measured using western blotting. The experiments were conducted with three independent replicates. G) GSEA of protein insertion into mitochondrial membrane involved in the apoptotic signaling pathway of embelin induced in NCI‐H2122 cells. NES > 1.5, *p *< 0.05, *q *< 0.25. H, I) NCI‐H2122 cells were treated with embelin (1, 10, and 15 µM) and SHP099 (10 µM) for 24 h, and MitoSOX (H) and MitoTracker (I) expressions were measured using an IF assay. The experiments were conducted with three independent replicates. (J, K) NCI‐H2122 cells were instantaneously transfected with si*PTPN11* and si*XIAP* for 24 h, individual or both, and ROS release and fluorescence intensity statistics J) and percentage of late apoptosis cells K) and were assesses, with siNC as the control. ^**^ *p *< 0.01 and ^***^ *p *< 0.001 compared to siCtrl group. The experiments were conducted with three independent replicates. L) TUNEL and DAPI fluorescence staining of tumor tissue in NCI‐H2122 subcutaneous transplantation tumor mouse model (× 200). ^**^ *p *< 0.01 compared to control or siCtrl group; ^***^ *p *< 0.001 compared to control or siCtrl group.

To further elucidate the antitumor effects of embelin, we performed transcriptomic analysis. Differential expression analysis of transcriptomics showed that embelin upregulated 1993 genes and suppressed 1825 genes compared to the control group (**Figure** **4**A). GO clustering analysis of the DEGs showed that embelin is involved in regulating cell motility, cell cycle, ERBB signaling pathway, cellular senescence, and ERK and MAPK pathways (Figure S10A, Supporting Information). KEGG enrichment analysis showed that embelin influenced metabolic processes such as carbohydrate metabolism, lipid, and amino acid metabolism, and interfered with cellular processes such as cell growth and death, cellular communication, transporter and catabolism, and cellular motility; regulated the endocrine, immune, nervous, and digestive system functions of the organism; and participated in therapeutics for viral infectious diseases, specific types of cancer, bacterial infectious diseases, neurodegenerative diseases, and immune diseases (Figure S10B, Supporting Information). We further generated a protein interactome based on the transcriptomic data and subdivided the list of proteins into subnetworks that were significantly enriched by the actions of embelin based on biological function. Consistent with the expected effects of embelin, we identified the regulation of pathways including apoptosis, cell cycle, and proliferation. Notably, we also identified multiple pathways previously overlooked with embelin, including RAS signaling pathway, and endoplasmic reticulum stress. In addition, it was shown that embelin is involved in kinase and phosphatase regulation (Figure S10C, Supporting Information).

GO enrichment analysis of DEGs in transcriptomics showed that embelin activated endogenous and exogenous apoptotic pathways and affected mitochondrial function and DNA fragmentation through apoptosis (Figure 4B). The apoptosis assay showed that embelin increased the proportion of early and late apoptotic cells in a concentration‐dependent manner and that SHP099 promoted apoptosis (Figure 4C; Figure S11A, Supporting Information). Elevated ROS levels may act as second messengers to upregulate pro‐apoptotic proteins, promote the opening of the mitochondrial permeability transition pore, activate caspases, and participate in the Ca^2+^ pathway to induce apoptotic cell death.^[^ ^31^, ^32^ ^]^ We examined the effect of embelin on ROS release from NCI‐H2122 cells, and the results showed that both embelin and SHP099 promoted ROS release in and embelin‐promoted ROS showed concentration‐dependent manner (Figure 4D). To further investigate the role of ROS release in the anticancer effects of embelin, two known ROS scavengers, n‐acetylcysteine (NAC), and butylated hydroxyanisole (BHA) were used to observe their effects on embelin‐induced antitumor efficacy at the cellular level. Our results showed that ROS scavengers partially attenuated the antitumor effect of embelin, indicating that ROS production mediates the anticancer effect of embelin (Figure S11B, Supporting Information). A hierarchical clustering heatmap of apoptosis differential genes showed that embelin significantly upregulated the expression of *PARP*, *CASP7*, and *TP53* and downregulated *BCL2* and *PIK3R3* (Figure 4E). Consistent with this, embelin increased the expression of cleaved caspase 3, cleaved caspase 7, cleaved PARP, Bid, Bak, and Bim, and decreased the expression of the apoptosis‐inhibiting protein BCL‐XL in NCI‐H2122 and A549 cells (Figure 4F; Figure S11C, Supporting Information). The Gene Set Enrichment Analysis (GSEA) of transcriptomic DEGs showed that protein insertion into the mitochondrial membrane is also involved in embelin‐regulated cellular processes, suggesting that embelin promotes endogenous apoptosis (Figure 4G). We then detected the effects of embelin on mitochondrial superoxide and mitochondrial membrane potential changes using MitoSOX and MitoTracker. The results showed that both embelin and SHP099 increased the expression of mitochondrial superoxide and decreased the mitochondrial membrane potential difference, suggesting that embelin promotes endogenous apoptosis (Figure 4H,I). Given the crucial role of cytochrome *c* release in the intrinsic mitochondrial‐mediated apoptosis pathway, its expression was assessed in both mitochondria and the cytoplasm. Embelin treatment led to cytochrome *c* release from mitochondria into the cytoplasm in A549 cells, suggesting activation of the mitochondrial apoptotic pathway (Figure S11D, Supporting Information). To investigate the mechanism through which embelin affects apoptosis, we examined the apoptotic ratio and ROS release after the knockdown of XIAP and SHP2. The results showed that the knockdown of XIAP and SHP2 alone had no effect on late apoptosis, but increased the proportion of prematurely apoptotic cells and promoted intracellular ROS release, and the combined effect also increased the proportion of prematurely apoptotic cells and ROS release (Figure 4J,K; Figure S11E, Supporting Information). Moreover, the knockdown of XIAP or SHP2 alone similarly induced cytochrome *c* release and the simultaneous knockdown of both proteins further amplified this effect. These results suggested that embelin induces mitochondrial‐mediated apoptosis by concurrently targeting both XIAP and SHP2 (Figure S11F, Supporting Information). In addition, TUNEL staining results showed that genomic DNA breaks increased and fluorescence staining was enhanced in tumor tissues after embelin and combination treatment, indicating that embelin induced apoptosis in tumor cells in vivo (Figure 4L). These results suggested that embelin induces apoptosis by targeting XIAP and SHP2.

### 2.5 Embelin Inhibits Cancer‐Related Signaling Pathways in *KRAS*‐Mutant NSCLC Cells

**Figure 5:**
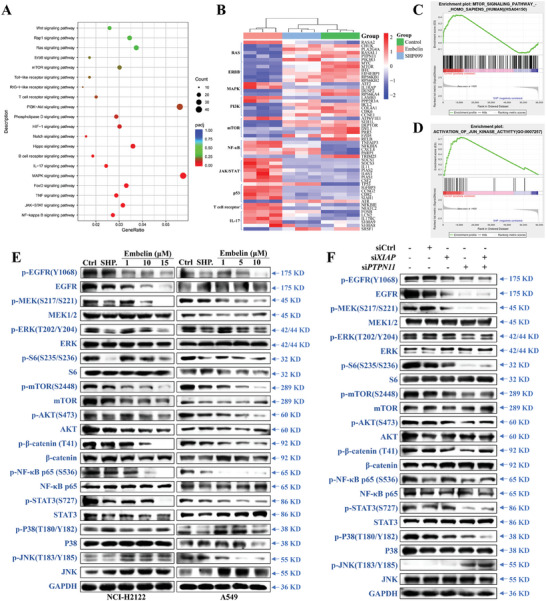
Embelin inhibits cancer‐related signaling pathways in *KRAS*‐mutant NSCLC cells. A) Dot map of KEGG enrichment analysis for the top 20 signaling pathways obtained through cluster analysis of DEGs between the control group and the embelin treated group in transcriptomics data. B) Heatmap hierarchical clustering displayed the DEGs in different signaling pathways between the control, SHP099, and embelin‐treated groups using RNAseq with *p*adj < 0.05. C, D) GSEA of activation of JUN kinase activity (C) and mTOR signaling pathway (D) of embelin induced in NCI‐H2122 cells. NES >1.5, *p *< 0.05, *q* < 0.25. E) Experiment validation of protein expression and phosphorylation of ERK/MAPK, p38/MAPK, JNK/MAPK, PI3K/AKT, Wnt, NF‐κB, and JAK/STAT signaling pathways in A549 and NCI‐H2122 cells after 24 h of treatment with various concentrations of embelin and SHP099. F) NCI‐H2122 cells were transfected with si*PTPN11* or si*XIAP* or both for 24 h, and changes in expression and phosphorylation of ERK/MAPK, p38/MAPK, JNK/MAPK, PI3K/AKT, Wnt, NF‐κB, and JAK/STAT signaling pathways were measured using western blotting. All western blot experiments were conducted with three independent replicates.

A dot graph of KEGG enrichment analysis of the top 20 signaling pathways obtained by clustering analysis of differential genes between the control and embelin‐treated groups in transcriptomic data showed that embelin primarily affected the signaling pathways of nuclear factor kappa B (NF‐κB), Janus kinase/signal transducers and activators of transcription (JAK/STAT), tumor necrosis factor (TNF), and MAPK in cells (**Figure** **5**A). To further explore the effects of embelin on signaling pathways, we visualized the genes with the most significant changes in each pathway in the NCI‐H2122 cells (Figure 5B). GSEA of DEGs revealed that embelin also affected JNK activity and the mTOR signaling pathway (Figure 5C,D). In addition, the inhibitory effects of embelin on RAS/MAPK, phosphoinositide‐3‐kinase (PI3K)/AKT, JAK/STAT, Wnt, and NF‐κB signaling pathways were validated by western blotting (Figure 5E). Embelin inhibited MEK and ERK phosphorylation in NCI‐H2122 and A549 cells, whereas SHP099 inhibited MEK and ERK phosphorylation in NCI‐H2122 cells but did not affect MEK phosphorylation in A549 cells (Figure 5E). Embelin and SHP099 inhibited the phosphorylation of S6, mTOR, and AKT in A549 and NCI‐H2122 cells, indicating that SHP2 phosphorylation was positively associated with the PI3K/AKT signaling pathway in *KRAS*‐mutant NSCLC cells (Figure 5E). In addition, the knockdown of XIAP and SHP2, either separately or in combination, significantly inhibited MEK phosphorylation and reduced the phosphorylation of AKT, mTOR, and S6 in NCI‐H2122 cells (Figure 5F). Notably, other XIAP inhibitors inhibited ERK and mTOR phosphorylation, but the Smac analogs had no effect on ERK, S6, or mTOR expression and phosphorylation (Figure S12A, Supporting Information). Other SHP2 inhibitors suppressed SHP2 and ERK phosphorylation at high concentrations (Figure S12B,C, Supporting Information). These results suggest that targeting XIAP and SHP2 inhibits the RAS/MAPK and RAS/PI3K signaling pathways. In addition, we found that embelin inhibited β‐catenin, NF‐κB p65, P38 phosphorylation and promoted JNK phosphorylation. In addition, SHP099 inhibited β‐catenin, NF‐κB p65, P38 phosphorylation in A549 cells (Figure 5E). Combined knockdown of XIAP and SHP2 decreased the levels of β‐catenin, P38, and NF‐κB p65 phosphorylation and increased the level of JNK phosphorylation in NCI‐H2122 cells. Knockdown of SHP2 alone inhibited β‐catenin, NF‐κB p65, and P38 phosphorylation, while promoting JNK phosphorylation, whereas XIAP knockdown alone inhibited the phosphorylation of β‐catenin and P38 phosphorylation, and promoted JNK phosphorylation (Figure 5F). Unlike its effects in A549 and NCI‐H2122 cells, embelin did not induce caspase‐3 activation and inhibit the RAS/MAPK or RAS/PI3K signaling pathways in KRAS wild‐type HCC827 and NCI‐H1975 cells (Figure S13, Supporting Information).

The above results suggest that embelin inhibits the RAS/MAPK, PI3K/AKT, JAK/STAT, Wnt, and NF‐κB signaling pathways in *KRAS*‐mutant NSCLC cells.

### 2.6 Embelin Interferes with SHP2 Signaling Complex Formation in *KRAS*‐Mutant NSCLC Cells

**Figure 6:**
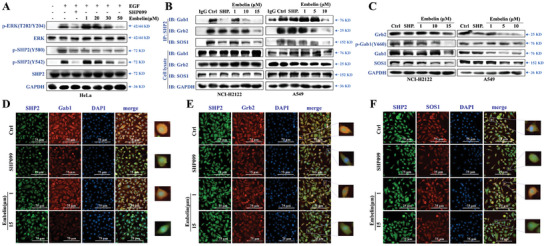
Embelin disrupts the SHP2 signaling complex in *KRAS*‐mutant NSCLC cells. A) HeLa cells were pretreated with SHP099 (20 µM) or different concentrations of embelin for 2 h and stimulated with EGF (10 ng mL^−1^) for 2 h, the protein expression of RAS/MAPK signaling pathway in HeLa cells was detected. B) After treatment of embelin and SHP099 (10 µM) in NCI‐H2122 and A549 cells for 12 h, cell lysates were collected. One part was used to measure Gab1, Grb2, and SOS1 expression, and the other part underwent IP with SHP2 antibody. Then, binding of Gab1, Grb2, and SOS1 with SHP2 was detected. C) After treatment of embelin and SHP099 (10 µM) for 24 h, the expression of Gab1, p‐Gab1, Grb2, and SOS1 proteins in A549 and NCI‐H2122 cells were detected. D, E, and F) NCI‐H2122 cells were treated with SHP099 (10 µM) and embelin (1 or 10 µM) for 12 h, then incubated with Gab1 and SHP2 antibodies (D), Grb2 and SHP2 antibodies (E), and SOS1 and SHP2 antibodies (F). Co‐localization of these proteins with SHP2 was detected using IF. All experiments were conducted with three independent replicates.

To further explore the mechanism of the signaling pathway influenced by embelin, we treated KRAS wild‐type HeLa cells with EGF and monitored the effect of embelin on the phosphorylation of SHP2. The results showed that EGF stimulation promoted the phosphorylation of SHP2 (Y542 and Y580) and increased ERK phosphorylation, whereas SHP099 or embelin pretreatment significantly inhibited these phosphorylation levels, suggesting that embelin may interfere with SHP2‐related complex formation (**Figure** **6**A). After 12 h of embelin treatment, the interaction between SHP2 and SOS1, Gab1, and Grb2 was attenuated in NCI‐H2122 cells, similar to that in SHP099 (Figure 6B). Western blotting revealed that SOS1, Grb2, and Gab1 protein expression and phosphorylation levels were reduced after embelin treatment in A549 and NCI‐H2122 cells (Figure 6C). In addition, colocalization of SHP2, SOS1, and Grb2 was significantly inhibited, and SOS1 and Grb2 expression was significantly reduced in NCI‐H2122 cells after embelin treatment for 24 h (Figure 6D–F). These results suggested that embelin interferes with SHP2 signaling complex formation in *KRAS*‐mutant NSCLC cells.

### 2.7 Embelin Inhibits Negative Feedback in *RAS* Mutant Cells

**Figure 7:**
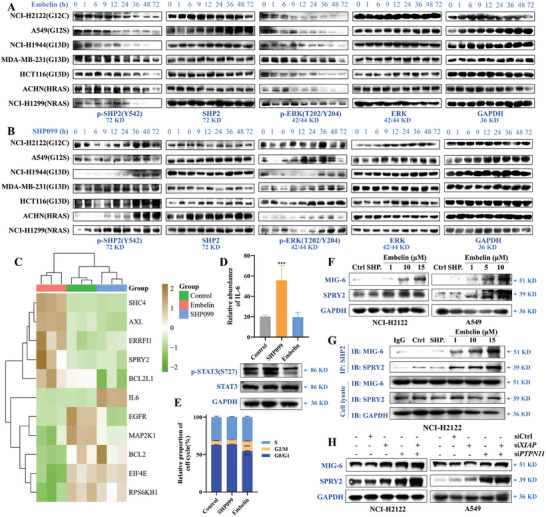
Embelin suppresses negative feedback in *RAS*‐mutant cells. A) Effects of embelin on SHP2 and ERK expression and phosphorylation in *KRAS‐, NRAS‐*, and *HRAS‐*mutant cells at different times. B) Effects of SHP099 on SHP2 and ERK expression and phosphorylation in *KRAS, NRAS*, and *HRAS‐*mutant cells at different times. C) Heatmap hierarchical clustering displays DEGs in KEGG pathway of EGFR tyrosine kinase inhibitor resistance between the control, SHP099, and embelin‐treated groups through transcriptomics with *p*adj < 0.05. D) Effects of embelin and SHP099 on IL‐6 mRNA expression and STAT3 phosphorylation level of NCI‐H2122 cells were assessed through transcriptomics detection and western blotting. (D) Statistical graph of cycle ratio of NCI‐H2122 cells after treating with embelin and SHP099 at 10 µM for 96 h. ^***^ *p *< 0.001 compared to control group (unpaired two‐tailed Student's *t*‐test). E) Statistical graph of cycle ratio of NCI‐H2122 cells after treating with embelin (15 µM) or SHP099(10 µM) for 72 h. ^***^ *p *< 0.001 compared to control group (unpaired two‐tailed Student's *t*‐test). F) Expression changes in MIG‐6 and SPRY2 in NCI‐H2122 and A549 cells after treatment with embelin and SHP099 (10 µM) for 24 h. G) Changes in MIG‐6 and SPRY2 combined with SHP2 in NCI‐H2122 cells treated with embelin (1, 10, and 15 µM) and SHP099 (10 µM) for 12 h. H) Expression changes in MIG‐6 and SPRY2 protein expression assessed using western blotting in NCI‐H2122 and A549 cells after transfection with si*PTPN11* and si*XIAP* separately or together for 24 h. ^***^ *p *< 0.001 compared to control group. All experiments were conducted with three independent replicates.

ERK phosphorylation levels are concomitant with SHP2 phosphorylation after long‐term application of TKIs as well as RAS/MAPK inhibitors.^[^ ^33^ ^]^ Compared with traditional SHP2 inhibitors, embelin inhibited the negative feedback occurring in *RAS*‐mutant lung cancer cells, which was manifested by the long‐term inhibition of SHP2 and ERK phosphorylation, whereas the levels of ERK and SHP2 phosphorylation were restored to some degree by the addition of SHP099 (**Figure** **7**A,B). To explore the molecular mechanism, hierarchical clustering heatmaps of DEGs in the KEGG pathway of EGFR tyrosine kinase inhibitor resistance by transcriptomics indicated that embelin promoted the expression of *SPRY2*, *AXL*, *BCL2L1*, and *ERRFI1* and downregulated *MAP2K1* and *BCL2*, whereas SHP099 promoted *IL‐6* and *EGFR* expression (Figure 7C). Consistent with the above findings, IL‐6 relative expression abundance and STAT3 phosphorylation levels were elevated in the SHP099 group after 72 h of continuous processing, whereas no change was detected in the embelin group (Figure 7D). Cell cycle experiments revealed that SHP099 had no effect, whereas embelin blocked the G2/M cycle (Figure 7E).

It has been shown that MIG‐6 and SPRY2 regulate RTK‐related pathways by inhibiting the RTK receptor itself or its downstream proteins.^[^ ^34^ ^]^ Overexpression of MIG‐6 and SPRY2 inhibited the phosphorylation of ERK, SHP2, and AKT, whereas their knockdown had the opposite effect, promoting the phosphorylation of these signaling molecules (Figure S14A,B, Supporting Information). These findings support the notion that MIG‐6 and SPRY2 act as negative regulators of the RAS signaling pathway. We then examined the effects of embelin and knockdown of *PTPN11* and *XIAP* on MIG‐6 and SPRY2 protein expression in NCI‐H2122 cells. The results showed that embelin significantly promoted the expression of MIG‐6 and SPRY2 in a concentration‐dependent manner and enhanced the interaction between SHP2 and these repressors (Figure 7F,G). In addition, the knockdown of *PTPN11* also promoted the expression of MIG‐6 and SPRY2, and XIAP knockdown enhanced these changes (Figure 7H). Moreover, to further investigate whether MIG‐6 and SPRY2 strengthen the negative feedback effect triggered by SHP099, or induce the feedback in embelin treated cells, MIG‐6 and SPRY2 were knocked down following treatment with embelin or SHP099 in A549 cells. Our results showed that the knockdown of MIG‐6 and SPRY2 significantly attenuated the inhibitory effects of embelin on ERK, SHP2, and AKT phosphorylation, suggesting that MIG‐6 and SPRY2 induce negative feedback regulation of RAS signaling inhibited by embelin (Figure S14C,D, Supporting Information). Consistent with this, we found knockdown of MIG‐6 and SPRY2 partly strengthened negative feedback resulting from SHP099, implying that MIG‐6 and SPRY2 increase negative feedback in SHP099 treated cells (Figure S14C,D Supporting Information).

These results indicate that SHP099 can generate RAS signaling feedback by upregulating IL‐6 expression and STAT3, whereas embelin can inhibit the compensatory effect of the RAS signaling pathway by upregulating the expression of MIG‐6 and SPRY2, and by increasing their interaction with SHP2.

## 3 Discussion

The treatment of *KRAS*‐mutant tumors encounters several obstacles, such as acquired drug resistance, tumor immune inertia, and apoptosis tolerance.^[^ ^1^, ^35^ ^]^ Hence, exploiting the concept of synthetic lethality is an alternative approach to increasing the vulnerability of cancer cells to oncogenic KRAS activation. We found that XIAP gene levels were significantly positively correlated with KRAS in both LUAD and LUSC tissues in the TCGA database and were significantly highly expressed in RAS mutant cells, particularly in *KRAS*‐mutant tumor cells, which is consistent with the observation that XIAP is upregulated by RAS and binds directly to C‐RAF kinase, a direct RAS effector protein that initiates the classical mitogen‐activated protein kinase (MAPK) cascade, to modulate its stability.^[^ ^36^, ^37^ ^]^ Higher expression of XIAP was found in lung cancer tumors, consistent with the observation that the oncogenic RAS pathway is activated in 84% of LUAD cases, including 65% of KRAS wild‐type tumors.^[^ ^38^ ^]^ Upregulation of XIAP expression induces resistance to TRAIL‐induced apoptosis in cancer cells. Therefore, XIAP may contribute to the insensitivity of SHP2 inhibitors to suppress lung cancer cell proliferation and induce apoptosis. This is supported by our observation that genetic knockdown or pharmacological inhibition of XIAP or SHP2 exhibited no obvious effect on lung cancer cells; however, combination treatment with XIAP and SHP2 inhibitors or embelin significantly suppressed cell proliferation, metastasis, and induction of apoptosis, demonstrating that XIAP and SHP2 are synthetic lethal partners in lung cancer with high RAS activity. Multiple studies in cultured cells have demonstrated that overexpression of XIAP confers resistance to multi‐agent chemotherapy, including stimulation of the mitochondrial and death receptor pathways of caspase activation, and that knocking out XIAP with siRNA or antisense oligonucleotides restores chemosensitivity to a variety of malignant cell lines.^[^ ^29^, ^39^ ^]^ Knockout of XIAP is not toxic to normal cells, as evidenced by the lack of significant pathology in XIAP‐knockout mice.^[^ ^40^, ^41^ ^]^ Chemotherapy remains the first‐line choice for *RAS*‐mutant LUAD. Hence, our results support the clinical use of both XIAP and SHP2 inhibitors in these patients, which may provide more potent anticancer effects and are worthy of further investigation.

Embelin, extracted from the fruits of *Embelia ribes*, is a benzoquinone alkaloid with a variety of medicinal properties, including antibacterial, antiparasitic, chemopreventive, antitumor, anti‐inflammatory, antioxidant, and hepatoprotective activities. It has potential clinical significance in a wide range of chronic diseases, such as autoimmune inflammatory diseases, diabetes mellitus, obesity, cardiovascular and cerebral vascular diseases, and cancer.^[^ ^42^, ^43^ ^]^ As a nonpeptide cell‐permeable inhibitor, embelin binds to the XIAP‐baculoviral IAP repeat domain (BIR) 3 structural domain with a binding affinity similar to that of the natural Smac peptide, thereby eliminating the apoptosis‐inhibitory effect induced by XIAP and exerting antitumor effects in vitro and in vivo.^[^ ^44^ ^]^ Combined XIAP inhibition with PPARγ or p53 activation, autophagy, or BCL2 inhibition has been found to enhance apoptosis in acute myeloid leukemia or ovarian granulosa cell tumors.^[^ ^28^, ^45^, ^46^ ^]^ Previous studies on embelin focused only on XIAP targets.^[^ ^47^ ^]^ Our experiments for the first time identified SHP2 as an additional target of embelin, which specifically inhibited SHP2 activity. Existing SHP2 enzymatic inhibitors primarily bind to active sites with strong polarity within the catalytic domain of SHP2 (e.g., C459 cysteine residue) via their intramolecular negatively charged phosphomimetic groups, resulting in the loss of activity. However, the high degree of homology between the catalytic domains of SHP2 and phosphatases, such as SHP1, has resulted in poor selectivity for most SHP2 inhibitors, and there is a lack of SHP2 inhibitors that balance selectivity and cell permeability.^[^ ^18^, ^31^, ^48^ ^]^ Currently, SHP2 allosteric inhibitors, such as SHP099 and RMC‐4550, selectively inhibit SHP2 by disrupting its autoinhibitory conformation by acting at the interface between the N‐terminal SH2, C‐terminal SH2, and the catalytic region of SHP2, generating hydrogen‐bonding interactions with key amino acid residues in the three structural domains (Arg^111^, Phe^113^, and Glu^250^), thereby inhibiting SHP2 activity.^[^ ^49^, ^50^ ^]^ During the screening of SHP2 allosteric inhibitors, the phosphorylated tyrosine IRS‐1 or Gab1 peptide was used to activate the full‐length SHP2 to mimic the opening of the N‐terminal SHP2 domain on the catalytic domain, and the SHP2 PTP domain was used to eliminate the enzymatic inhibitors.^[^ ^51^, ^52^ ^]^ This was based on the hypothesis that there are no allosteric sites in the PTP domain. In this study, we showed that embelin specifically inhibited the activity of SHP2 without inhibiting the activity induced by Gab1 activated full‐length SHP2, thus challenging this hypothesis. The existence of novel allosteric sites in the PTP domain is also supported by the finding that the SHP2 catalytic domain contains a hidden allosteric site that does not exist in most other PTP family members and that covalent inhibition of Cys^333^ in the PTP domain could achieve specific inhibition of SHP2 activity. By comparing the amino acid sequence and surface structure of the PTP structural domain with those of SHP1 (PDBID:4GRY) and PTP1B (PDBID:8G69), we found that His^114^ was not conserved in PTPs and that the PTP pocket region of SHP2 had a significantly larger spatial structure, which provided a structural basis for the affinity of embelin with SHP2. In addition, Glu^250^ and Arg^111^ were the two major amino acids that SHP099 interacts with to achieve allosteric inhibition. We also compared the predicted embelin binding sites in SHP2 with other PTPs and found that Lys^325^ is not conserved in other PTPs, which provided a basis for specific inhibition of embelin on SHP2. Consistent with the effects of SHP099, embelin inhibited SHP2 and ERK phosphorylation in *KRAS*‐mutant and wild‐type cells. Moreover, embelin suppressed intracellular SHP2 activity and interfered with the interaction of SHP2 with adaptors Gab1, Grb2, and SOS1 in KRAS‐mutant cells, indicating that embelin affects enzymatic and nonenzymatic functions by orienting SHP2. Although small‐molecule compounds that stabilize SHP2 autoinhibition (e.g., SHP099) hold great promise for targeting cancers involving SHP2, the inhibitory activity of these compounds against cancer‐associated SHP2‐activating mutants is greatly diminished because mutations in SHP2 disrupt their autoinhibitory conformation. However, compounds that inhibit PTP activity by acting directly on the allosteric site of the PTP structural domain of SHP2 could provide an alternative mode of allosteric SHP2 targeting. Therefore, it is of interest to further elucidate the exact binding mode of embelin to SHP2, which will be helpful in identifying novel allosteric sites in the PTP domain for drug discovery.

Deletion or inhibition of SHP2 in established tumors delays tumor progression but is not sufficient to achieve tumor regression, whereas simultaneously inhibiting XIAP expression using siRNA technology can inhibit tumor cell proliferation.^[^ ^53^, ^54^ ^]^ The anticancer effects of SHP2 and XIAP inhibition were consistent with our observation that embelin inhibited the proliferation of different *KRAS*, *NRAS*, *HRAS*‐mutant, and *KRAS* wild‐type cells in vitro and in vivo by reducing the expression of Ki67, PCNA, and intracellular ATP, with relatively specific inhibition on the proliferation of *KRAS* mutant cells. Previous studies have shown that NSCLC cells exhibit senescence‐like behaviors after knockdown of *PTPN11* in a low‐serum medium, and inhibition of the XIAP gene increases senescent cell death by attenuating the binding of autophagic vesicles to lysosomes, thereby inhibiting the downstream process of autophagy.^[^ ^55^, ^56^ ^]^ This is consistent with our finding that embelin and SHP099 induced elevated β‐galactosidase expression and a senescent phenotype. In addition, we found that SHP2 and XIAP inhibitors alone had little effect on cell proliferation or intracellular ATP content; however, their combined effect resulted in a significant decrease in cell proliferation and intracellular ATP content. Therefore, the promotion of senescent cell death may be responsible for the induction of the synthetic lethality of SHP2 and XIAP at the animal and cellular levels. SHP2 participated in the regulation of TGF‐β1‐mediated EMT in A549 cells, and targeting SHP2 may reverse EMT in *KRAS*‐mutant NSCLC.^[^ ^57^ ^]^ Consistent with this, the SHP2 inhibitor, SHP099, partially reversed EMT and reduced the malignant phenotype of *KRAS*‐mutant cells, whereas embelin reduced the migratory and invasive abilities, expression of mesenchymal marker proteins, and increased epithelial cell‐specific proteins in a concentration‐dependent manner. In contrast to the observation that XIAP deletion increased cell migration and motility, we showed that knockdown of XIAP resulted in increased expression of E‐cadherin and decreased expression of vimentin and fibronectin, indicating that XIAP is positively correlated with EMT in *RAS*‐mutant tumor cells. In addition, the knockdown of both XIAP and SHP2 reduced the migration and invasion abilities of cancer cells and decreased the expression of EMT marker proteins. mTOR regulates EMT in prostate cancer through the RhoA and Rac1 pathways.^[^ ^58^ ^]^ Transcriptomic analysis and omics validation showed that mTOR expression was reduced by both SHP099 and embelin, explaining the reversal of EMT at the molecular level. These results suggest that targeting XIAP with SHP2 reduces the cellular metastatic ability by reversing EMT, thereby reducing the malignant phenotype of the cells. The BIR3 domain in the protein structure of XIAP binds to the apoptosis‐initiating protein Caspase‐9, and BIR2 domain acts on the effector proteins caspase‐3 and caspase‐7, thus inhibiting the catalytic activity of caspases and exerting antiapoptotic effects, which may contribute to the ability of embelin to induce apoptosis in cells.^[^ ^24^, ^59^ ^]^ XIAP amplifies FAS/cd9‐mediated cell death signaling via Bid‐dependent mitochondrial outer membrane potential.^[^ ^60^ ^]^ Moreover, SHP2 can modulate the mitochondrial membrane potential and the subsequent release of ROS by interacting with adenine nucleotide translocase 1 (ANT1), the most abundant protein in the mitochondria, which is central to mitochondrial integrity.^[^ ^61^ ^]^ Therefore, the dual targeting of XIAP and SHP2 may synergistically disturb mitochondrial homeostasis to induce apoptosis.

SHP2 participates in various signaling processes, including those initiated by growth factors such as platelet‐derived growth factor (PDGF) and epidermal growth factor (EGF), and cytokines such as GM‐CSF, EPO, insulin, and interferon. XIAP‐selective antagonists strongly inhibit NOD2‐mediated activation of NF‐κB and MAPK signaling and subsequent cytokine or chemokine production.^[^ ^24^, ^62^ ^]^ We showed that embelin or knockdown of SHP2 and XIAP inhibited the JAK/STAT, MAPK, PI3K/AKT, NF‐κB, and Wnt signaling pathways. In response to growth factors, SHP2 is recruited to the phosphorylated tyrosine sites on reactive RTKs and/or binds to the scaffold adapter via the SH2 domain, thereby becoming activated.^[^ ^63^, ^64^ ^]^ Consistent with this, we found that SHP2 and its downstream effector molecule ERK were largely unphosphorylated, whereas phosphorylation levels were upregulated by EGF treatment, and pretreatment with embelin or SHP099 reversed the above changes in HeLa cells with *KRAS*‐wild type genotype. An inhibitory effect of embelin and SHP099 on SHP2 and ERK phosphorylation was observed in *KRAS*‐mutant cells, suggesting that targeting SHP2 alters its own phosphorylation state, which in turn affects downstream signaling pathways. In particular, activated RTKs phosphorylate the C‐terminal tyrosine residues (Tyr542 and Tyr580) of SHP2, forming binding sites for various intracellular proteins, such as Gab1, Grb2, JAK2, and IRS‐1, thus connecting RTKs with multiple signaling pathways.^[^ ^65^, ^66^, ^67^, ^68^ ^]^ In addition, we also found that embelin attenuated the interaction between SHP2 and adaptor proteins, such as SOS1, Gab1, and Grb2 after a short period of action, and reduced the expression of SHP2 junction proteins after prolonged action, whereas SHP099 yielded less effect on Grb2. All the above results point to the possibility that embelin inhibits multiple signaling pathways partly by interfering with SHP2/Gab1/Grb2/SOS1 complex formation. Furthermore, we discovered that embelin inhibited the negative feedback in multiple RAS‐mutant cells compared to that in SHP099. MIG‐6 and SPRY2 are one class of tumor suppressor genes (TSGs), that play crucial roles in modulating RTKs through feedback loops.^[^ ^34^ ^]^ Direct MIG‐6‐EGFR interaction promotes receptor endocytosis and degradation.^[^ ^69^ ^]^ The interaction of MIG‐6 with signaling molecules such as GRB2 and PI3Kp85 generally affects RTK signaling, leading to the inhibition of downstream pathways such as RAS/MAPK/ERK and PI3K/AKT.^[^ ^70^, ^71^ ^]^ SPRY2 has two facets of RTKs signaling pathway regulation, which depend on the cellular state and balance. The most prominent inhibitory activity of SPRY2 stems from its ability to interact with downstream signaling molecules, including FRS2, SHP2, GRB2, RAF, and PLCδ, centered on the RAS/RAF/MAPK pathway.^[^ ^72^, ^73^ ^]^ However, its interaction with the casitas B‐lineage lymphoma proto‐oncogene (CBL) E3 ubiquitin ligase may positively regulate EGFR signaling because this interaction isolates CBL and blocks CBL‐mediated EGFR degradation.^[^ ^74^, ^75^ ^]^ We also found that the short duration of embelin enhanced the interaction of SHP2 with MIG‐6 and SPRY2, and its prolonged action increased the expression of MIG‐6 and SPRY2 in cells, whereas SHP099 did not, which explains why embelin does not generate negative feedback in the MAPK pathway and thus addresses the poor clinical efficacy of SHP2 allosteric inhibitors. It has been shown to SHP2 promotes SPRY2 mRNA expression and inhibits its activity by dephosphorylating SPRY2 during the post‐translational phase in lens and lacrimal gland development; however, there is no such evidence in tumor cells so far. We discovered that the expression of MIG‐6 and SPYR2 was also increased by knockdown of XIAP and SHP2, indicating that targeting XIAP and SHP2 can inhibit negative feedback in cells by enhancing the expression of inhibitory factors, such as MIG‐6 and SPRY2, providing direct evidence linking XIAP and SHP2 to MIG‐6 and SPRY2 for the first time. Recent studies have shown that IL‐6 expression is upregulated in *EGFR*‐mutated NSCLC tumors, which mediates resistance to EGFR‐TKIs.^[^ ^76^ ^]^ This was also observed in our transcriptomics and validation results, accompanied by enhanced phosphorylation of STAT3 downstream of IL‐6 in SHP099 treatment, which further expanded the indications for dual targeting of XIAP and SHP2.

In summary, SHP2 is an intracellular target of embelin that can reduce the malignant phenotype of KRAS‐mutant NSCLC in vivo and ex vivo by co‐targeting XIAP and SHP2 to inhibit multiple cancer‐related pathways to reverse EMT, overcome the negative feedback of the MAPK signaling pathway, and induce apoptosis in tumor cells, thus providing a novel approach to address the apoptosis tolerance and adaptive resistance of *KRAS*‐mutant tumors. Our results revealed that SHP2 and XIAP are potential synthetic lethal partners, and embelin provides the backbone for the development of novel SHP2/XIAP dual‐targeting inhibitors to yield innovative therapeutic strategies for the alleviation of *KRAS*‐mutant NSCLC.

## 4 Experimental Section

### PTPs Activity Assay

Catalytic activity screening methods for PTPs using candidate compounds were performed as previously described.^[^ ^77^ ^]^ In brief, soluble fusion PTP proteins, His‐ΔSHP2, encompassing the peptide from amino acids 205–593 retaining PTP activity, His‐ΔSHP2^K325A^, His‐ΔSHP1, encompassing the peptide from amino acids 202–595 retaining PTP activity, His‐PTP1B, His‐HePTP, His‐VHR, and His‐TCPTP were cloned, expressed, and purified in *E. coli*. After determining protein concentration using bicinchoninic acid (BCA) assay kit, 1 µg fusion protein, 10 µM library compound were added in 100 µL reaction mixture (55 mM 4‐(2‐hydroxyethyl)‐1‐piperazineethanesulfonic acid [HEPES], 100 mM sodium chloride [NaCl], 0.5 mM ethylenediaminetetraacetic acid [EDTA], 1 mM dithiothreitol [DTT], 0.01% Triton X‐100, 0.002% bovine serum albumin [BSA], and 0.1% dimethyl sulfoxide [DMSO]) and incubated at 37 °C for 30 min in the dark, with Na_3_VO_4_ as positive control. The detection substrate DiFMUP (50 µM) was promptly added to each reaction and functioned at 37 °C for 30 min in the dark. The fluorescence intensity was then measured using Multimode Plate Reader (λa = 358 nm, λe = 455 nm, PerkinElmer, USA).

For detection of SHP2 allosteric regulation by the compounds, the soluble fusion His‐SHP2 protein was cloned, expressed, and purified by constructing a pET28a‐*PTPN11* recombinant plasmid in *E. coli*. Subsequently, 148 nM His‐SHP2 protein with 14.8 µM peptide (bis‐phosphorylated peptide from Gab1, with the sequence of: H2N‐VE(pY)LDLDLD(PEG8)RVD‐(pY)VVVDQQ‐amide) was mixed thoroughly in reaction buffer and incubated for 30 min at 37 °C in the dark. The fluorescence intensity was detected by DiFMUP after addition of different drugs and incubation at 37 °C for 30 min, with SHP099 and Na_3_VO_4_ as positive controls and unactivated protein as negative controls.

To test intracellular SHP2 activity, the extract of NCI‐H2122 cells was precleared with protein A/G agarose beads and incubated with anti‐SHP2 antibody overnight at 4 °C, followed by further incubation with protein A/G agarose beads for 2 h at 4 °C. The Iimmunoprecipitates were suspended in sample buffer and detected in 96‐well black polystyrene plates using the fluorescent substrate DiFMUP under the same conditions as described above.

### Isothermal Titration Calorimetry (ITC)

The purified His‐ΔSHP2, His‐SHP2, or His‐ΔSHP2^K325A^ proteins were dialyzed overnight in dialysis buffer (20 mM HEPES, 0.15 m NaCl, 1 mM TCEP, 10% glycerol, pH 7.5). The protein concentration was determined using the BCA Protein Assay Kit, and the purity of the proteins was detected by Coomassie Brilliant Blue staining. ITC experiments were performed when the protein concentration was higher than 2 mg mL^−1^ and the purity was greater than 90%. The instrument (Malvern Instruments, USA) was considered to be functioning optimally for compound and protein titration experiments when the DP value was < 0.05 µcal s^−1^ by the water droplet experiment. After the sample cell was cleaned twice with protein dialysis buffer, a protein sample (300 µL) was loaded to the sample cell, an equal amount of ultrapure water was added to the reference cell, and the compound solution dissolved with the same dialysis buffer was put into the corresponding cell, in which the protein‐compound molar ratio was 1:10–1:20. The parameters were adjusted based on the curve‐fitting results to determine the optimal binding concentration gradient. Based on the exothermic changes in the reaction, the entropy increase and enthalpy change were analyzed using software fitting to obtain parameters such as the binding coefficient and affinity constant between the small molecules and proteins.

### Site‐Directed Mutation Assay

A single‐point mutation assay of the pET32a‐ΔSHP2 plasmid was performed using the QuickMutation Site‐Directed Mutagenesis Kit (Beyotime, Shanghai, China). The relevant mutation primers are as follows: ΔSHP2^K325A^‐F: 5′‐AACAATTCAAAGCCCAAAGCGAGTTACATTGCCACACAA‐3′; ΔSHP2^K325A^‐R: 5′‐TTGTGTGGCAATGTAACTCGCTTTGGGCTTTGAATTGTT‐3′. In brief, plasmids with mutation sites were produced by PCR amplification via primers containing mutation sites, and screened using DpnI enzyme to specifically digest methylated template plasmids; and the mutation results were verified through transformation and sequencing. The identified plasmids were then transformed into *E. coli*. BL21 competent cells for protein purification and expression. The recombinant protein His‐ΔSHP2^K325A^ was used for subsequent SHP2 activity assay and ITC.

### Reversibility Assessments of Embelin‐SHP2 Binding

To test whether embelin and SHP2 were reversibly bound, the purified His‐ΔSHP2 fusion protein was dialyzed overnight in dialysis buffer (10 mM HEPES, 0.5 m NaCl, 1 mM TCEP, and 10% glycerol, pH 7.5), and the protein activity was detected by DiFMUP. After incubating embelin with dialyzed protein at 37 °C for 30 min, the mixed solution was dialyzed overnight or ultrafiltered, with DMSO as a control, and protein activity was detected by DiFMUP to determine the mode of protein‐compound binding.

### Enzymatic Inhibition Analysis of SHP2

A Michaelis‐Menten model and Lineweaver‐Burk plot of SHP2 inhibition by embelin were established. In brief, enzyme kinetics of SHP2 varied with the concentrations of substrate DiFMUP and embelin, concentrations of embelin were selected at 0, 10, and 20 µM, and concentrations of DiFMUP were selected at 10, 20, 40, 80, and 160 µM. Recombinant His‐ΔSHP2 was preincubated for 30 min with embelin or DMSO at various concentrations in the absence of substrate and then assayed for enzymatic activity at different times as mentioned above.

### RNA Interference and Overexpression Assay

NCI‐H2122 or A549 cells in the logarithmic growth phase were collected and inoculated into 96‐well plates or six‐well plates, and cell transfection was performed when cell fusion reached 60%. Prepare transfection solution (200 µL well^−1^, 6‐well plate) according to the instructions of Transintro EL transfection reagent, which contained opti‐MEM medium (200 µL), siRNA or recombinant plasmid (50 nM), Transintro EL transfection reagent (9 µL), and 96‐well plate (10 µL well^−1^) was added in above reagents with the same ratio. The reagent was mixed at room temperature for 15–20 min.

The siRNA was used to reduce the protein expression, the sequence of which was shown as below (si*XIAP*‐1 sense: 5′‐CUACUACACAGGUAUUGGUTT‐3′, si*XIAP*‐1 antisense: 5′‐ACCAAUACCUGUGUAGUAGTT‐3′; si*XIAP*‐2 sense: 5′‐CAUGGAUAUACUCAGUUAATT‐3′, si*XIAP*‐2 antisense: 5′‐UUAACUGAGUAUUACCAUGTT‐3′; si*PTPN11*‐1 sense: 5′‐GCAAUGACGGCAAGUCUAATT‐3′, si*PTPN11*‐1 antisense: 5′‐UUAGACUUGCCGUCAUUGCTT‐3′; si*PTPN11*‐2 sense: 5′‐GCAAUGACGGCAAGUCUAATT‐3′; si*PTPN11*‐2 antisense: 5′‐UUAGACUUGCCGUCAUUGCTT‐3′; si*ERRFI1*‐1 sense: 5′‐GGUGUGGACAAAUCAACUA‐3′, si*ERRFI1*‐1 antisense: 5′‐UAGUUGAUUUGUCCACACC‐3′; si*ERRFI1*‐2 sense: 5′‐GGAGAUCAGAGUCCCAUUA‐3′, si*ERRFI1*‐2 antisense: 5′‐UAAUGGGACUCUGAUCUCC‐3′; si*SPRY2*‐1 sense: 5′‐GUCUCACUGUUGUACACGA‐3′, si*SPRY2*‐1 antisense: 5′‐UCGUGUACAACAGUGAGAC‐3′; si*SPRY2*‐2 sense: 5′‐GGUCCAUUCUUCUGCACGA‐3′, si*SPRY2*‐2 antisense: 5′‐UCGUGCAGAAGAAUGGACC‐3′). Transfection of the recombinant plasmid, p3×Flag CMV‐7.1‐*PTPN11*, pCMV3‐*ERRFI1*, and pCMV3‐*SPRY2* were used to overexpress SHP2, MIG‐6, and SPRY2. When the transfection was finished, the plates were incubated at 37 °C for a specific time period for subsequent reactive oxygen species (ROS) release, adenosine triphosphate (ATP) release, colony formation, western blotting, or the CCK‐8 assay.

### Cellular Thermal Shift Assay (CETSA)

NCI‐H2122 cells were harvested and resuspended with pre‐cooled PBS containing 1% cocktail, which was then frozen in liquid nitrogen for 5 min and thawed in a water bath at 37 °C for 5 min for three repetitions. The supernatant was collected via centrifugation at 4 °C (15 000 × g, 20 min), divided into two equal portions; one was added with test drugs or various concentrations of embelin, and the other was added with DMSO as control and subsequently incubated at 25 °C for 30 min. All samples were heated at temperatures ranging from 52–70 °C for 3 min, and subsequently in an ice bath for 3 min and collected by centrifugation at 4 °C (15 000 × g, 20 min), and added with protein sampling buffer at 100 °C for 5 min, followed by analysis of protein stability using western blotting.

### Co‐IP Assay

After treating NCI‐H2122 or A549 cells with given processing, the cells were lysed using pre‐cooled IP lysate (25 mM Tris HCl pH 7.4, 150 mM NaCl, 1 mM EDTA, 1% NP‐40, 1% PMSF and 5% glycerol), and centrifuged at 4 °C (14 000 rpm for 20 min) to collect the supernatant and then the protein concentration was determined using a BCA protein assay kit. The lysate and anti‐SHP2 antibody or other antibodies were added to a microcentrifuge tube and spun overnight at 4 °C to allow antigen‐antibody binding. And then protein A/G plus‐agarose beads were added to the tube and spun at 4 °C overnight. The magnetic beads were washed three times with IP lysate at 4 °C for 5 min and protein sampling buffer was added to the beads in a metal bath at 100 °C for 5 min, and the protein binding was analyzed using immunoblotting using the corresponding antibodies.

### MitoSOX Assay

The cells were pre‐placed in cell culture plates, and moistened with culture medium, NCI‐H2122 cells were inoculated into 24‐well plates, cultured overnight, and treated with the appropriate drugs. MitoSOX solution was added and incubated for 30 min at 37 °C. Subsequently, the cells were washed with pre‐warmed phosphate‐buffered saline (PBS) and sealed with an antifluorescence quenching sealer containing DAPI in the dark for 30 min at room temperature. Images were captured using a High Content Imaging System (PerkinElmer, USA).

### MitoTracker Assay

The cell slides were pre‐placed in a cell culture plate, rinsed thoroughly with culture medium, and then inoculated NCI‐H2122 cells into a 24‐well plate for overnight culture. After specific drug treatment, MitoTracker Red CMXRos working solution was added and incubated at 37 °C for 30 min. After gently washing with preheated PBS, a sealing agent containing DAPI to prevent fluorescence quenching was added and reacted at room temperature in the dark for 30 min. Images were acquired using a High Content Imaging System (PerkinElmer, USA).

### Establishment of Subcutaneous Transplantation Tumor Model in Mice

All mouse experiments were conducted according to the animal protocols approved by the Institutional Animal Care and Use Committee.

NCI‐H2122 cells in the logarithmic growth phase were collected by mixing serum‐free RMPI‐1640 medium and Matrigel matrix gel, and injected subcutaneously into the right axilla of thymus‐less male BALB/c‐nu nude mice. Tumor volume was calculated according to V = ab^2^/2 (where a and b are the long and short diameters of the tumor, respectively), and body weight was recorded every 3 days. When the tumor volume reached 100 mm^3^, the mice were randomly divided into six groups (ten mice in each group): Control group (i.p, q2d), SHP099 (20 mg kg^−1^, i.p, q2d) group, GDC‐0152 (20 mg kg^−1^, i.p, q2d) group, SHP099 (20 mg kg^−1^, i.p, q2d) + GDC‐ 0152 (20 mg kg^−1^, i.p, q2d) co‐administration group, embelin (20 mg kg^−1^, i.p, q2d) medium‐dose group and embelin (30 mg kg^−1^, i.p, q2d) high‐dose group. When the tumor volume reached 1000 mm^3^, all experimental mice were sacrificed, and tumor tissues and major organs, such as the heart, liver, spleen, lungs, and kidneys, were collected for further study.

### Hematoxylin‐Eosin (H&E) Staining

The primary organs and tumor tissues of the mice were fixed with 4% tissue fixative overnight, rinsed with distilled water, dehydrated with a gradient alcohol solution, made transparent with xylene alcohol‐xylene‐paraffin xylene solution, and embedded in soft and hard paraffin. The paraffin blocks were sliced using a sectioning machine, spread using a spreading machine, and slid using a slide spreader. The slides containing the tissue sections were baked in a constant temperature oven at 62 °C (Yihang Technology, DHP‐9052) for 2 h. After gradient dewaxing with xylene alcohol‐distilled water, the sections were stained with a hematoxylin staining solution for 8 min, differentiated with 0.1% hydrochloric acid in alcohol for 6 s, rinsed for 15 min, stained with an eosin staining solution for 4 min, sealed with neutral gum, and observed under an inverted microscope.

### Immunohistochemical (IHC) Staining

After dewaxing in water, paraffin sections were subjected to antigen repair by adding 0.1 m sodium citrate buffer (pH 6.0) in an autoclave, inactivated peroxidase with 3% H_2_O_2_, and blocked with 5% BSA, and incubated overnight at 4 °C with drops of primary antibody. The samples were treated with droplets of HRP‐labeled anti‐rabbit/mouse antibodies for 30 min and then incubated with droplets of 3,3′‐diaminobenzidine (DAB) solution for 5–10 min at room temperature. The samples were re‐stained with hematoxylin for 2–3 min, rinsed with distilled water, and hydrated using a gradient of alcoholic xylene. The sections were then sealed with neutral gum and observed under an inverted microscope for protein expression and staining.

### Terminal Deoxynucleotidyl Transferase dUTP Nick End Labeling (TUNEL) Staining

After dewaxed to water, paraffin sections were incubated with DNase‐free proteinase K solution (20 µg mL^−1^) at 37 °C for 30 min, and washed with PBS three times. TUNEL staining solution (containing TdT enzyme, fluorescent labeling solution, and TUNEL detection solution) was added dropwise to the samples and incubated at 37 °C for 60 min under light protection, after which the slices were sealed with an antifluorescent quenching sealing solution containing DAPI. The cells were observed using a fluorescence microscope.

### Transcriptomics Detection and Analysis

After treatment with embelin and SHP099 for 24 h, RNA in NCI‐H2122 cells was extracted using TRIzol, and the concentration was detected using a NanoPhotometer, purity using a spectrophotometer, and integrity using an Agilent 2100 bioanalyzer. The mRNA was enriched using oligo (dT) magnetic beads and randomly interrupted with divalent cations. Oligonucleotides were designed as primers for cDNA synthesis and polymerase chain reaction (PCR) amplification to obtain cDNA libraries. Four kinds of fluorescent‐labeled dNTP, DNA polymerase, and splicing primers were added to the sequencing pool for amplification, and the cDNA library was converted into a sequencing library through computer software by the principle of synthesis and sequencing at the same time. Using the principle of sequencing during synthesis, optical signals were converted into sequencing peaks using computer software to obtain the sequence information of the fragments to be tested. Statistical analysis of differential genes was performed using the DESeq2 software; clusterProfiler R which was used for Gene Ontology (GO) enrichment analysis and statistical analysis of differentially expressed genes (DEGs) in the Kyoto Encyclopedia of Genes and Genomes (KEGG) pathway.

### Molecular Docking

AutoDock software was used for the flexible docking of small molecules and proteins. Molecular docking of candidate compounds was performed using the crystal structures of SHP2 full‐length region (PDBID: 5EHR), SHP2 catalytic region (PDBID: 7PPL), and ΔSHP2 region (PDBID: 6CMP, without N‐SH2 structural domains) obtained from the Protein Data Bank, respectively. Molecular docking and specific detection of embelin was performed by using the crystal structures of CDC25A (PDBID: 1C25), CDC25B (PDBID: 1QB0), DUSP6 (PDBID: 1MKP), LMPTP (PDBID: 7KH8), PRL‐1 (PDBID: 5BX1), PRL‐3 (PDBID: 1V3A), PTP1B (PDBID: 8G69), PTPN22 (PDBID: 3H2X), RPRPδ (PDBID: 4BPC), ΔSHP1 (PDBID: 4GRY), STEP (PDBID: 2BIJ), VEPTP (PDBID: 2HC2), XIAP‐BIR3 (PDBID: 4KMP). The specific operations are as follows: The planar structure of the small molecules was obtained using ChemDraw software, and the stereo structure was obtained using Chem3D software. Using AutoDockTools, the protein crystal structure was dehydrogenated, the charge was calculated, and the atom type was set The small molecules were dehydrogenated, the charge was adjusted, and the root was judged, generating a file that could be recognized by AutodockVina1.1. Semiempirical free‐energy calculations were used to assess binding between small molecules and proteins, and simulated annealing and genetic algorithms were used to determine the optimal binding sites. Data were visualized using the PyMOL and LigPlus software.

### Bioinformatics Analysis

Gene expression data for normal and tumor tissues were obtained from The Cancer Genome Atlas (TCGA) database (https://portal.gdc.cancer.gov/), and the data were analyzed and visualized using the ggplot2 package in R (version 3.6.3). The gene expression profiling database GEPIA2 (http://gepia2.cancer‐pku.cn) was used to analyze the relationship between *PTPN11* expression and the prognosis of patients with lung adenocarcinoma (LUAD) and to analyze the correlation between candidate compound genes and *KRAS* in the TCGA database. TIMER 2.0 (http://timer.comp‐genomics.org/timer/) was used to analyze the expression of candidate target genes in the TCGA database. Protein‐protein interaction (PPI) networks were constructed using the Search Tool for the Retrieval of Interacting Genes (STRING) online tool (https://cn.string‐db.org/) and Cytoscape (version 3.10.0, https://cytoscape.org/). Graphs and heatmaps for KEGG and GO analyses were created using the Microbiology Letters (http://www.bioinformatics.com.cn/) and Chiplot (https://www.chiplot.online/) online tools.

### Statistical Analysis

All experiments were repeated three times and data were expressed as Mean ± standard deviation (SD), analyzed, and plotted using SPSS 23.0 and GraphPad prism 6.2.1. Comparisons between two samples were made using Student's *t*‐test, and comparisons between multiple groups were tested for significance using one‐way ANOVA and the SNK (Newman) Keuls method, with differences considered statistically significant at *p* < 0.05.

The Z’ factor and signal‐to‐noise ratio (S/N) were calculated using the following equations:

(1) $$Z^{\prime }=1-\frac{3\times \left(\mathrm{SDsignal}\;+\;\mathrm{SDbackground}\right)}{\mathrm{Msignal}\;-\;\mathrm{Mbackground}}$$

(2) $$\frac{S}{N}=\frac{\mathrm{Msignal}\;-\;\mathrm{Mbackground}}{\sqrt{{\left(\mathrm{SDsignal}\right)}^{2}+{\left(\mathrm{SDbackground}\right)}^{2}}}$$

where SDsignal is the standard deviation of the positive control signal, SDbackground is the standard deviation of the negative control, Msignal is the mean of the positive control signal, and Mbackground is the mean of the negative control signal.

### Ethics Statement

The animal experiments were conducted in accordance with the guidelines adopted by the Chengdu Institute of Biology, Chinese Academy of Sciences. All mouse experiments were conducted according to the animal protocols approved by the Institutional Animal Care and Use Committee.

## Conflict of Interest

The authors declare no conflict of interest.

## Author Contributions

N.J.F. and Y.W.S. contributed equally to this work. F.W. and G.L.Z. supervised the study. N.J.F., F.W., and G.L.Z. designed the overall experiments. N.J.F., Y.W.S., Z.F., Z.W., L.Y.L., R.Y.X., and X.K.S. performed the biological experiments. N.J.F. and Z.F. performed the animal‐associated experiments. N.J.F. and Z.F. conducted the molecular docking and bioinformatics analyses. N.J.F. and Z.F. performed statistical analyses. F. W., G.L.Z., and N.J.F. wrote the manuscript.

## Supporting information



Supporting Information

Supplementary Table1

## Data Availability

All datasets used and/or analysed during the current study are available from the corresponding author on reasonable request. The RNA‐seq (GSA‐Human ID: HRA008456) data are available at the National Genomics Data Center (NGDC) at https://ngdc.cncb.ac.cn/gsa‐human.
