# Supplementary material for: Synthetic Lethality of SHP2 and XIAP Suppresses Proliferation and Metastasis in KRAS‐mutant Nonsmall Cell Lung Cancer
Source: Adv Sci (Weinh). 2025 Feb 24;12(15):2411642. doi: 10.1002/advs.202411642 (PMC12005787; doi:10.1002/advs.202411642)
Supplement: Supplementary file 1 — Supporting Information [file ADVS-12-2411642-s001.pdf]

ADVANCED SCIENCE
Open Access

# Supporting Information

for *Adv. Sci.*, DOI 10.1002/advs.202411642

Synthetic Lethality of SHP2 and XIAP Suppresses Proliferation and Metastasis in *KRAS*-mutant Nonsmall Cell Lung Cancer

*Nai-jie Fu, Yu-wen Sheng, Zhe Fan, Zhao Wu, Ling-yu Li, Rui-ying Xi, Xiao-ke Shi, Guo-lin Zhang and Fei Wang**

Supporting Information

# Synthetic Lethality of SHP2 and XIAP Suppresses Proliferation and Metastasis in *KRAS*-mutant Non-Small Cell Lung Cancer

*Nai-jie Fu[#], Yu-wen Sheng[#], Zhe Fan, Zhao Wu, Ling-yu Li, Rui-ying Xi, Xiao-ke Shi, Guo-lin Zhang, Fei Wang**

## 1. Experimental Section/Methods

### Experimental cells and animals

Human *KRAS*-mutant NSCLC cell lines NCI-H2122, A549, NCI-H1944, and NCI-H358; *KRAS*-mutant breast cancer cell line MDA-MB-231; *NRAS*-mutant NSCLC cell line NCI-H1299; and *HRAS*-mutant renal cancer cell line ACHN were obtained from Procell Life Science&Technology Co.,Ltd.(Wuhan, China). The *KRAS*-mutant colon cancer cell line HCT116 and *KRAS* wild-type (WT) cervical cancer cell line HeLa were obtained from the Shanghai Life Sciences Cell Bank of the Chinese Academy of Sciences (Shanghai, China). The *KRAS*-mutant colorectal cancer cell lines SNU-C2A, SW620, SW1116 and HCT15; *KRAS*-mutant lung cancer cell line NCI-H1155; *KRAS* WT lung cancer cell lines NCI-H1693 and NCI-H1755; and *HRAS*-mutant lung cancer cell line NCI-H1915 were obtained from American Type Culture Collection (Virginia, USA). The *KRAS*-mutant lung cancer cell line HCC44; *KRAS* WT lung cancer cell line NCI-H838; and *KRAS* WT colorectal cancer cell line SW1417 were obtained from Cobioer Biosciences CO.,Ltd (Nanjing, China). The *KRAS* WT lung cancer cell line HCC827 and NCI-H1975 were obtained from Procell Life Science & Technology Co. Ltd. (Wuhan, China). All the cell lines were identified by short tandem repeat (STR) analysis and cultured in RPMI-1640, Ham's F-12K, L-15, Dulbecco's modified Eagle medium (DMEM) or ACL-4 medium supplemented with 10% fetal bovine serum (FBS) and 1% penicillin/streptomycin (P/S) at 37 °C in a humidified incubator with 5% $CO_2$, respectively.

Specific pathogen free (SPF) BALB/c-nu immunodeficient mice (male, 5 weeks old, weighing 18–20 g) were purchased from Beijing Huafukang Laboratory Animal Technology Co Ltd (Beijing, China) and raised in a SPF feeding animal facility on a 12-h light/dark cycle at 25 °C and 55% humidity at animal laboratory of Chengdu Institute of Biology, Chinese Academy of Sciences (Chengdu, China). All experimental procedures were approved by the Experimental Animal Center of the Chengdu Institute of Biology and complied with humane care according to the 3R principle [License No.: SCXK (Beijing) 2019-0008].

### Experimental reagents

All reagents used in this experiment were obtained from commercial sources. All compounds and drugs are listed in Supplementary Table S1. All reagents used for cell culture are listed in Supplementary Table S2. The reagents required for immunofluorescence (IF) and co-immunoprecipitation (co-IP) are listed in Supplementary Table S3. The reagents used in animal experiments are listed in Supplementary Table S4. The western blot experimental

supplies are listed in Supplementary Table S5. The relevant reagents for protein extraction from *Escherichia coli* are listed in Supplementary Table S6. Inhibitory effects of embelin on *KRAS*-mutant and wild-type cells are listed in Supplementary Table S7.

**Cell viability assay**

For the adherent cell viability assay, cells in the logarithmic growth phase were seeded in 96-well culture plates at a density of $5\times10^3$ $well^{-1}$ and incubated overnight. Diluted compounds or DMSO were added, or specific treatments were incubated at 37 °C and 5% $CO_2$ for 24 h or 48 h. Cell viability was determined using CCK-8 or CellLight™ Cell Viability Assay Kit, according to the manufacturer's protocol. The luminescence signals of the treated samples were normalized to those of the DMSO group.

For spherical cell viability assays, cells in the logarithmic growth phase were inoculated at a density of $4\times10^3$ $well^{-1}$ into 96-well spherical cell microplates and incubated at 37 °C, 5% $CO_2$ for 24 h or 48 h. Cell viability was measured using the CellTiter-Glo® Luminescent Cell Viability 3D Cell Viability Assay Kit, according to the manufacturer’s protocol. The remaining steps were the same as those described above.

**Colony formation experiment**

Cells in the logarithmic growth phase were digested thoroughly into a single dispersed state and inoculated into six-well plates at a density of 100 $well^{-1}$. Subsequently, different concentrations of the drugs were added for 14 d, consecutively, and the solution was changed every 2 d. Next, the cells were fixed with 4% formaldehyde, stained with 0.1% crystal violet, and the number of clones was counted using an inverted microscope.

**Cell senescence β-galactosidase staining**

NCI-H2122, A549, and NCI-H1944 cells in the logarithmic growth phase were seeded into 48-well plates and cultured overnight. The supernatant was aspirated and fresh serum-free (0%), low-serum (3%), or regular serum (10%) medium with different drugs was added for 48 h. When the supernatant was discarded, β-galactosidase staining fixation fluid were added for 15 min and β-galactosidase staining solution were imported overnight at 37 °C. Finally, the staining results were recorded using an inverted microscope.

**Cell scratch assay**

NCI-H2122 and A549 cells were seeded in six-well plates. When the cell fusion reached 80%, the fused cells were scratched vertically along a straightedge with the tip of a 100 μL pipette, and washed with phosphate-buffered saline (PBS). And then the appropriate medium and drugs were added for an additional 24 h at 37 °C in a humid incubator. Finally, the scratch was photographed using an inverted microscope and the wound healing status was recorded.

**Cell migration assay**

NCI-H2122 and A549 cells were pre-starved in serum-free medium for 24 h and resuspended in serum-free medium containing 5 mg $mL^{-1}$ BSA. The cell suspension was added to Transwell chambers at a density of $5\times10^5$ $mL^{-1}$, and RPMI-1640 or Ham F-12K medium containing 10% FBS was added to the bottom of the Transwell chambers. Subsequently, drugs were added to the chambers and incubated at 37 °C for 48 h and cells were then fixed with 4% neutral formaldehyde for 40 min and stained with 0.1% crystal violet for 60 min. The chamber was washed with triple-distilled water, and images were collected using an inverted microscope.

**Cell invasion assay**

The Transwell was pre-coated with Matrigel matrix gel (50 μL $well^{-1}$) for 4 h, and the cell density was adjusted to $1.0\times10^5$ $mL^{-1}$, which was subsequently added to the chamber, and RPMI-1640 or Ham′ F-12K medium containing 10% FBS was added to the bottom of the Transwell, and the subsequent experiments were performed as described in *Cell migration assay*.

**Western blotting**

After given treatment, cells were lysed by ice bath with radio-immunoprecipitation assay (RIPA) buffer containing 1% phenylmethylsulphonyl fluoride (PMSF), and the supernatant was collected by centrifugation, and the protein concentration was subsequently determined using a BCA Protein Assay Kit. Depending on the molecular weight of the proteins, different concentrations of sodium dodecyl-sulfate polyacrylamide gel electrophoresis (SDS-PAGE) separator gels were prepared for protein electrophoresis. Equal amounts of proteins were sampled and subjected to SDS-PAGE gel electrophoresis; at the end of electrophoresis, the proteins were transferred to nitrocellulose (NC) membranes. The NC membrane was blocked with 5% skim milk and covered with a specific primary antibody overnight at 4 °C, in which the primary antibody was diluted at a ratio of 1:500–1:5000. The NC membrane was

incubated with a horseradish peroxidase (HRP)-conjugated secondary antibody at room temperature for 2 h and then incubated with enhanced chemiluminescence (ECL) solutions for 15 s at room temperature. A fully automated gel imaging system (General Electric Company, USA) was used for the exposure imaging.

**Confocal microscopy**

NCI-H2122 cells were inoculated into 96-well plates specialized for high-content cell imaging, treated with various drugs, fixed with 4% neutral formaldehyde, and sealed with 5% BSA after permeabilization with 0.05% Triton X-100. Subsequently, the cells were incubated with the given primary antibody overnight at 4 °C and then with corresponding fluorescent secondary antibody in dark at room temperature for 1 h. 4', 6-diamino-2-phenylindole (DAPI) was applied to nuclear staining. Fluorescence images were captured using a high-content analyzer (Thermo Scientific, USA).

**Apoptosis detection**

NCI-H2122 cells treated with the given disposal were digested with trypsin, resuspended in cold PBS, and centrifuged at room temperature (1000×g for 5 min) to discard the supernatant. Approximately 100,000 cells were collected and 195 μL of Annexin V-FITC conjugate was added to gently resuspend the cells and then 5 μL of Annexin V-FITC and 10 μL of propidium iodide (PI) staining solution were added respectively. They were then incubated at 37 °C for 10-20 min in the dark, placed on ice, and passed through a flow cytometer (Becton, Dickinson, Calibur, USA) for online detection.

**Cell cycle assay**

NCI-H2122 cells treated with given disposal were washed with pre-cooled PBS, fixed with ice pre-cooled 70% ethanol at 4°C for 2 h. And then PI staining solution (containing RNaseA) was incubated in dark at 37°C for 30 min. Red fluorescence and light scattering were detected by flow cytometry at 488 nm. Cell cycle results were analyzed and visualized using ModFit LT and FlowJo software.

**Reactive Oxygen Species (ROS) Release Assay**

After specific treatment of NCI-H2122 cells, ROSup was added to the blank well for 30 min before detection as a positive control. Original medium discarded, and cells were washed with PBS, then serum-free cell culture medium was added and incubated with

Dichlorofluorescein diacetate (DCFH-DA) probe at 37°C for 30 min in an incubator. The intracellular ROS levels were detected by flow cytometry (λa = 488 nm, λe = 525 nm).

**Immunofluorescence (IF)**

For cellular experiments, the cell crawls preplaced in cell culture plates, NCI-H2122 and A549 cells were cultured into 24-well plates overnight, and then treated with the given drugs. Subsequently, cells were fixed with 4% neutral formaldehyde, washed with TBST, and incubated with 5% BSA. The primary antibody was added 4°C overnight and followed by fluorescent secondary antibody for 1 h away from light at room temperature. The slices were sealed with a DAPI-containing anti-fluorescence quenching sealer for 30 min at room temperature and images were captured using a High Content Imaging System (PerkinElmer, USA).

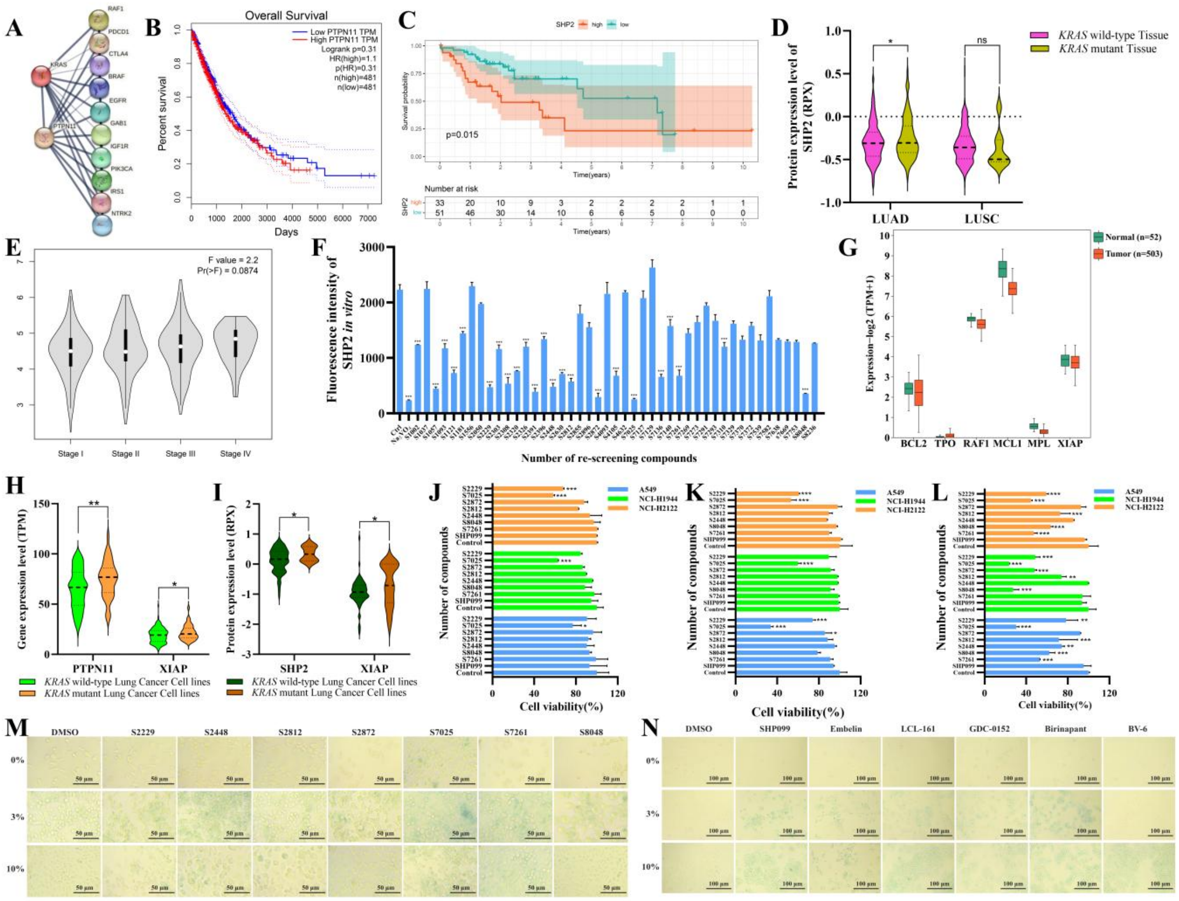


**Figure S1. Identification of embelin as an inhibitor against *KRAS*-mutant tumor by targeting SHP2 and XIAP.** (A) PPIs related to *KRAS* and *PTPN11* obtained through the STRING database. (B) Correlation of *PTPN11* expression with the OS of patients with LUAD (*n*=481) in the TCGA database. (C) Correlation and number at risk of SHP2 expression with the overall survival probability of *KRAS* mutant LUAD patients (*n* = 84) in the TCGA database. (D) The protein expression of SHP2 in *KRAS* mutant LUAD (*n* = 85), wild-type LUAD (*n* = 269), *KRAS* mutant LUSC (*n* = 8) and wild-type LUSC (*n* = 310) tissues $^{*}p < 0.05$ compared to *KRAS* wild-type tissue (unpaired two-tailed Mann–Whitney u test). (E) Correlation between the high expression of *PTPN11* and clinical stages of patients with LUAD. (F) Re-screening results of compounds with high inhibitory activity $^{***}p < 0.001$ compared to control (unpaired two-tailed Student's *t*-test). The experiments were conducted with three independent replicates. (G) *BCL2*, *TPO*, *RAF1*, *MCL1*, *MPL,* and *XIAP* mRNA expression levels in LUSC tumors and adjacent tissues through TCGA database. (H) The gene expressions of *PTPN11* and *XIAP* in *KRAS* mutant (*n* = 37) and wild-type (*n* = 140) lung cancer cell lines in the CCLE database. $^{*}p < 0.05$ and $^{**}p < 0.01$ compared to *KRAS* wild-type group (unpaired two-tailed Mann–Whitney u test). (I) The protein expressions of SHP2 and XIAP in *KRAS* mutant (*n* = 14) and wild-type (*n* = 85) NSCLC cell lines in the HPA database.

(J, K, and L) Effect of candidate drugs at 1 (J), 10 (K), and 50 μM (L) on cell viability in A549, NCI-H2122, and NCI-H1944 cells. $^{**}p < 0.01$, $^{***}p < 0.001$ and $^{***}p < 0.0001$ compared to control (unpaired two-tailed Student's *t*-test). The experiments were conducted with three independent replicates. (M) Effects of candidate drugs (10 μM) on cell senescence by β-galactosidase staining in NCI-H2122 cells. (N) Effect of SHP099, embelin, and different XIAP inhibitors on cell senescence of NCI-H2122 cells. The experiments were conducted with three independent replicates.

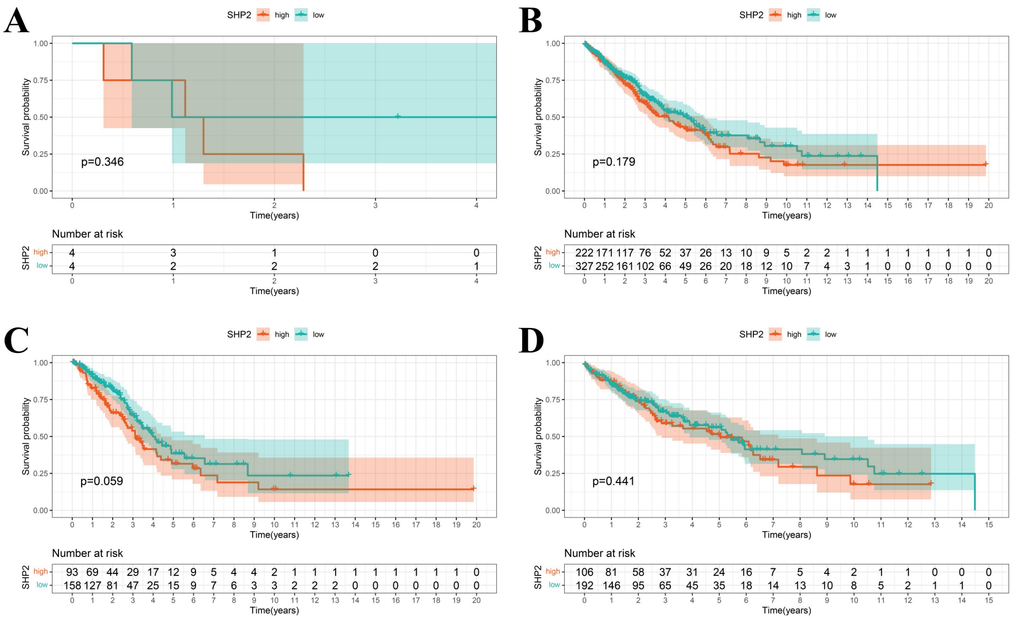


**Figure S2. Effect of high or low SHP2 expression on overall survival probability in *KRAS* wild-type and mutant lung cancer.** (A) Correlation and number at risk of SHP2 expression with the OS probability of *KRAS* mutant LUSC patients ($n$ = 8) in the TCGA database. (B) Correlation and number at risk of SHP2 expression with the overall survival probability of *KRAS* wild-type lung cancer patients ($n$ = 549) in the TCGA database. (C) Correlation and number at risk of SHP2 expression with the overall survival probability of *KRAS* wild-type LUAD patients ($n$ = 251) in the TCGA database. (D) Correlation and number at risk of SHP2 expression with the overall survival probability of *KRAS* wild-type LUSC patients ($n$ = 298) in the TCGA database.

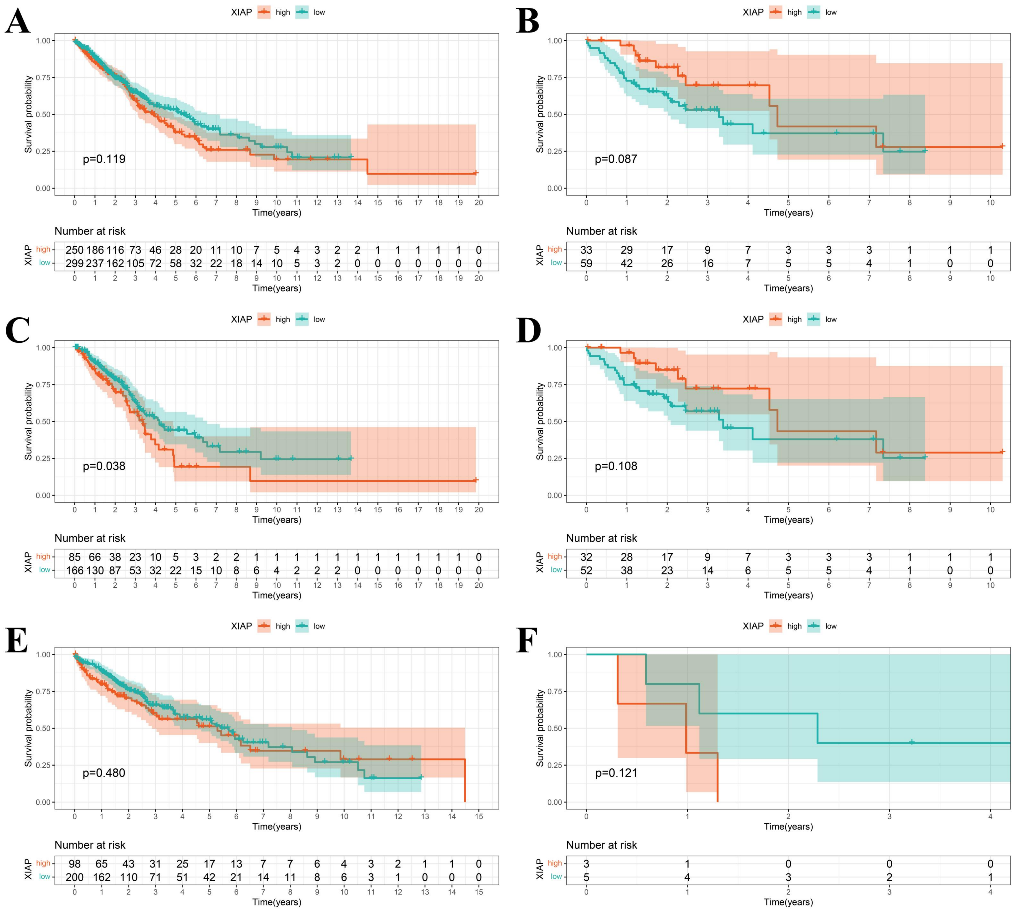


**Figure S3. Effect of high or low XIAP expression on OS probability in *KRAS* wild-type and mutant lung cancer.** (A) Correlation and number at risk of XIAP expression with the overall survival probability of *KRAS* wild-type lung cancer patients ($n$ = 549) in the TCGA database. (B) Correlation and number at risk of XIAP expression with the overall survival probability of *KRAS* mutant lung cancer patients ($n$ = 92) in the TCGA database. (C) Correlation and number at risk of XIAP expression with the overall survival probability of *KRAS* wild-type LUAD patients ($n$ = 251) in the TCGA database. (D) Correlation and number at risk of XIAP expression with the overall survival probability of *KRAS* mutant LUAD patients ($n$ = 84) in the TCGA database. (E) Correlation and number at risk of XIAP expression with the overall survival probability of *KRAS* wild-type LUSC patients ($n$ = 298) in the TCGA database. (F) Correlation and number at risk of XIAP expression with the overall survival probability of *KRAS* mutant LUSC patients ($n$ = 8) in the TCGA database.

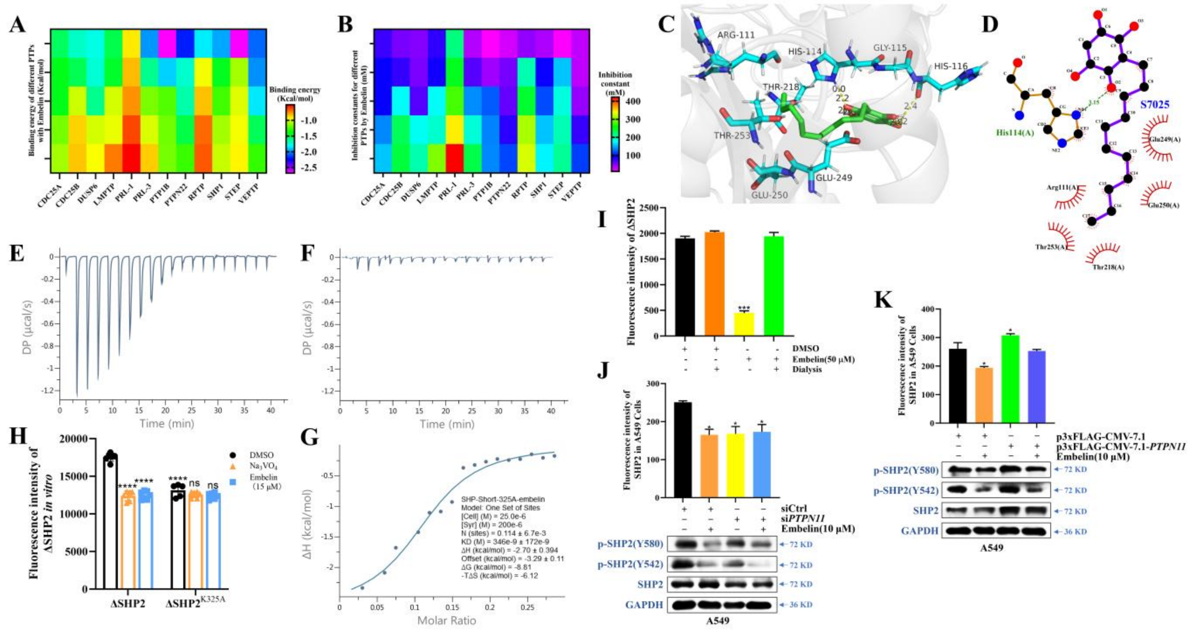

**Figure S4. Identification of embelin as an inhibitor of SHP2.** (A) Heatmap of top five conformations binding energy of the docking with different PTPs with embelin. (B) Heatmap of top five conformations inhibition constants of the docking with different PTPs with embelin. (C, D) Embelin was docked to full-length SHP2 (C). Important binding residues and amino acids are shown (D). (E) ITC titration results of embelin and His-ΔSHP2. (F, G) ITC titration results (F) and ITC data fitting diagram of 1:1 binding mode (G) of embelin and His-ΔSHP2$^{K325A}$. (H) The effect of embelin on enzyme activity of His-ΔSHP2 and His-ΔSHP2$^{K325A}$, with $Na_3VO_4$ as positive control. $^{****}p < 0.0001$ compared to DMSO+His-ΔSHP2 group; $^{ns}\ p > 0.05$ compared to DMSO+ΔSHP2$^{K325A}$ group (unpaired two-tailed Student's *t*-test). The experiments were conducted with three independent replicates. (I) Changes of His-ΔSHP2 activity after co-incubation and dialysis with embelin. $^{***}p < 0.001$ compared to DMSO group (unpaired two-tailed Student's *t*-test). The experiments were conducted with three independent replicates. (J) A549 cells were transfected with si*PTPN11* for 24 h and embelin was added for 24 h. Expression and phosphorylation of SHP2 in cell lysates were detected using western blotting and catalytic activity of SHP2 were tested using co-IP and fluorescence reading. $^{*}p < 0.05$ compared to *si*Ctrl (unpaired two-tailed Student's *t*-test). The experiments were conducted with three independent replicates. (K) p3×Flag-CMV-7.1-SHP2 was transfected into A549 cells for 24 h, then embelin at 10 μM was added for 24 h. Expression and phosphorylation of SHP2 in cell lysates were detected using western blotting and catalytic activity of SHP2 were tested using co-IP and fluorescence reading. $^{*}p < 0.05$ compared to p3xFLAG-CMV-7.1 group (unpaired two-tailed Student's *t*-test). The experiments were conducted with three independent replicates.

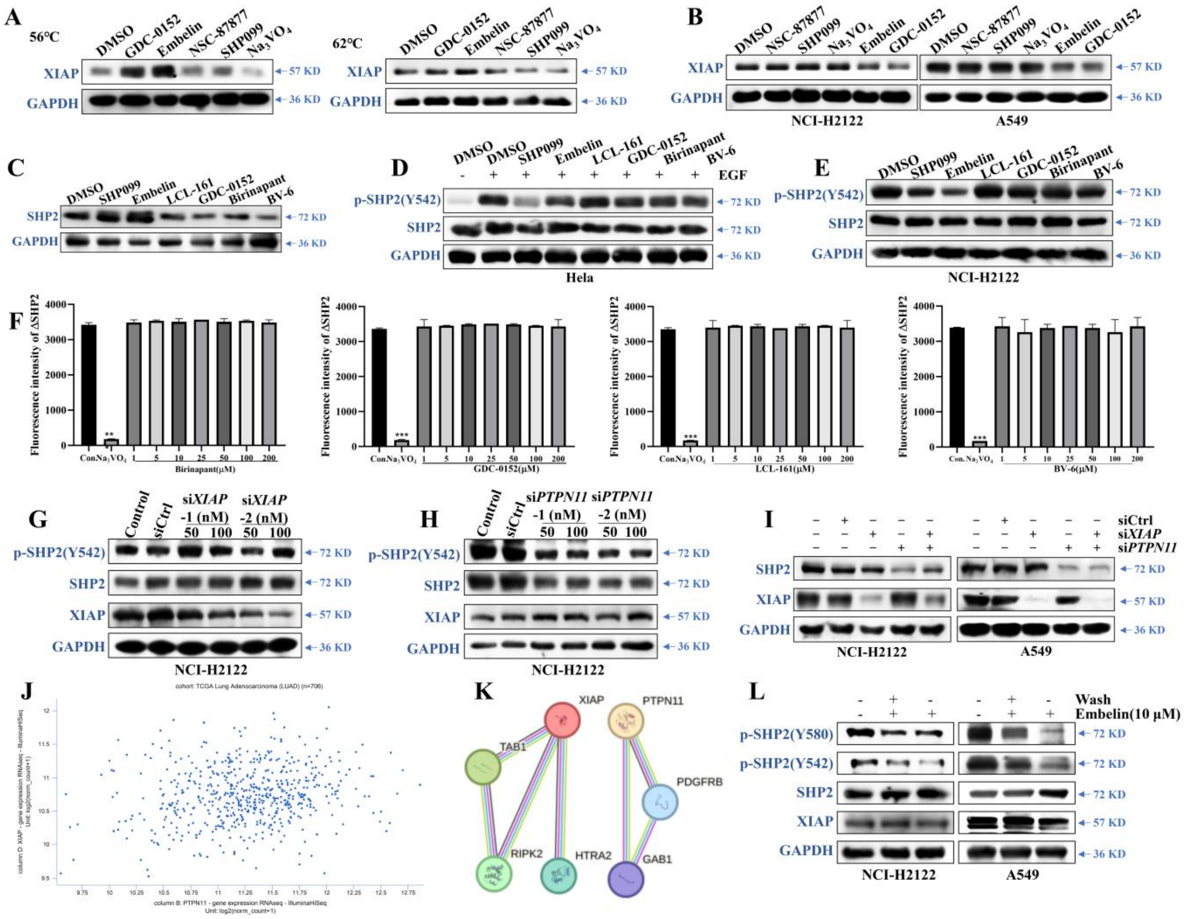


**Figure S5. Effects of XIAP and SHP2 on expression, phosphorylation, or activity of one another.** (A) Detection of XIAP protein binding with different SHP2 inhibitors at 56 °C (left) and 62 °C (right) using the CETSA assay. (B) Effect of SHP2 inhibitors on XIAP expression in NCI-H2122 and A549 cells measured using western blotting. (C) Detection of SHP2 protein binding with different XIAP inhibitors using the CETSA assay. (D) HeLa cells were pretreated with DMSO, SHP099, or various compounds at 20 μM for 2 h before stimulation with EGF (10 ng $mL^{-1}$). Western blotting was performed with SHP2, p-SHP2, and GAPDH antibodies. (E) Effect of different XIAP inhibitors on SHP2 expression in NCI-H2122 cells using western blotting. (F) Effect of different XIAP inhibitors on His-ΔSHP2 activity. $^{*}p <$ 0.05 compared to control group (unpaired two-tailed Student's *t*-test). (G) Effects of si*XIAP* on XIAP expression, SHP2 expression, and phosphorylation in NCI-H2122 cells after transient transfection for 24 h. (H) Effects of si*PTPN11* on XIAP expression, SHP2 expression, and phosphorylation in NCI-H2122 cells after transient transfection for 24 h. (I) Expression changes of XIAP and SHP2 expression in NCI-H2122 and A549 cells after transfection with si*PTPN11* and si*XIAP* separately or together for 24 h. (J) Correlation detection between mRNA expression of PTPN11 and XIAP in patients with LUAD ($n$ = 706) through IlluminaHiSeq analysis using data derived from TCGA database. (K) PPIs related to

*XIAP* and *PTPN11* from the STRING database. (L) Effects of embelin treatment for continuous 24 h or removal for 18 h after embelin incubation for 6 h on XIAP expression, SHP2, expression and phosphorylation in A549 and NCI-H2122 cells. All experiments were conducted with three independent replicates.

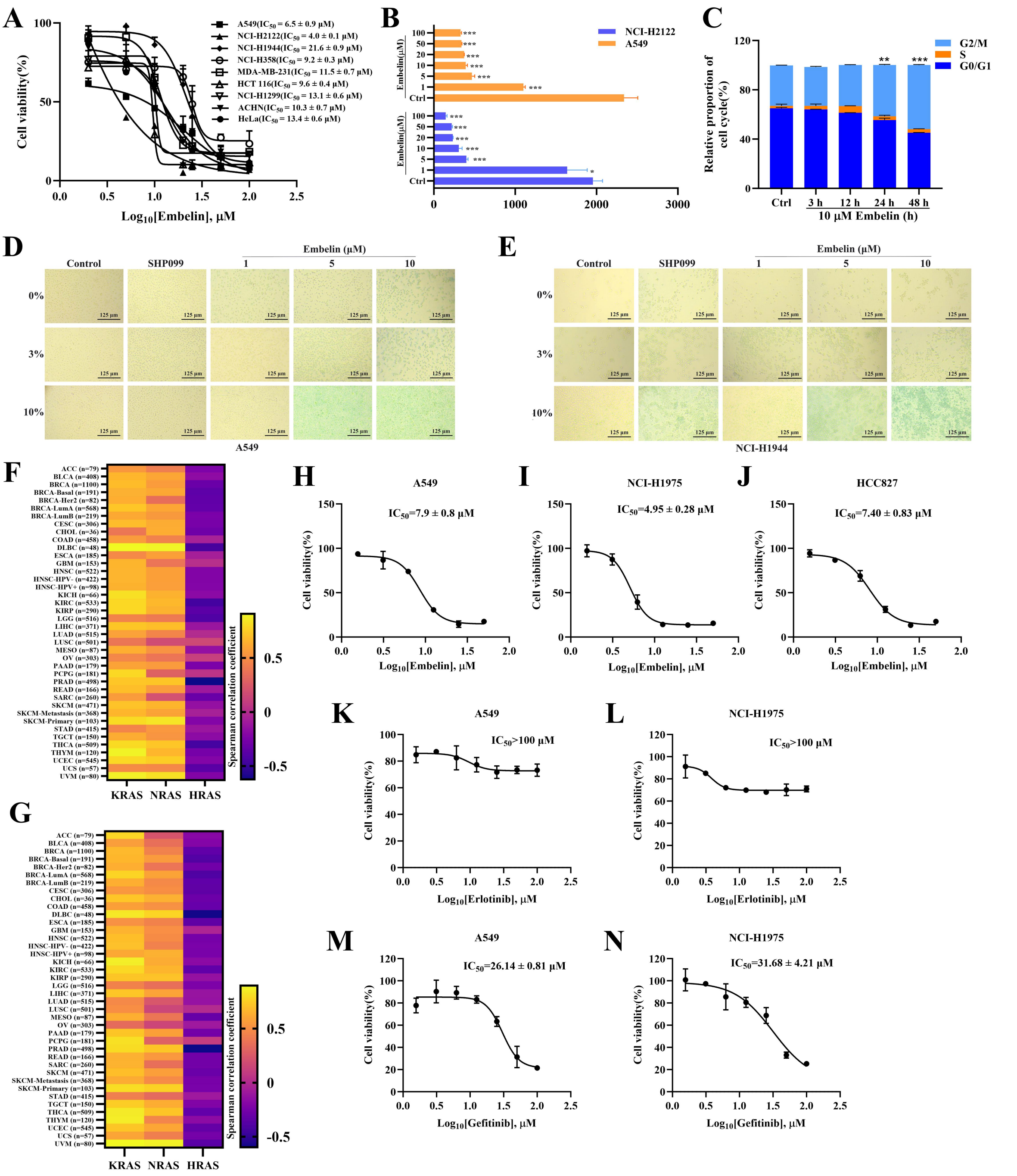


**Figure S6. Embelin inhibits cell proliferation and migration in *KRAS*-mutant NSCLC cells.** (A) Effect of embelin on cell viability across a mini-panel of *KRAS*-mutant and *KRAS*-wild type cell lines measured using the CCK-8 for 48 h. Bars, ± SEM. The curves were plotted using a variable slope (four-parameter) non-linear fit. (B) Cells were treated with embelin for 24 h in single-layer cell mode, the intracellular ATP chemiluminescence were detected. $^{*}p < 0.05$ and $^{***}p < 0.001$ compared to control group (unpaired two-tailed Student's *t*-test). (C) Statistical graph of cycle ratio of NCI-H2122 cells after different treatment times at 15 μM of embelin. $^{**}p < 0.01$ and $^{***}p < 0.001$ compared to control group (one-way ANOVA).

(D, E) Effects of embelin and SHP099 inducing senescence by β-galactosidase staining in human A549 (D) and NCI-H1944 (E) cells. The above experiments were conducted with three independent replicates. (F) Spearman's correlation coefficient between *PTPN11* and *KRAS*, *NRAS,* and *HRAS* mRNA in different types of tumors through TCGA database. (G) Spearman's correlation coefficient between *XIAP* and *KRAS* as well as *NRAS* and *HRAS* mRNA in different types of tumors through TCGA database. (H, I and J) The effect of embelin on cell viability of *KRAS*-mutant A549 (H), *EGFR*-mutant NCI-H1975 (I) and *KRAS* wild-type HCC827 (J) cell lines after 24 h were measured using the CCK-8. (K, L) The effect of EGFR-TKI erlotinib on cell viability of *KRAS*-mutant A549 (K) and *EGFR*-mutant NCI-H1975 (L) cell lines after 24 h were measured using the CCK-8. (M, N) The effect of EGFR-TKI gefitinib on cell viability of *KRAS*-mutant A549 (M) and *EGFR*-mutant NCI-H1975 (N) cell lines after 24 h were measured using the CCK-8. Bars, ± SEM. The curves were plotted using a variable slope (four-parameter) non-linear fit. Bars, ± SEM. The curves were plotted using a variable slope (four-parameter) non-linear fit. The experiments were conducted with three independent replicates.

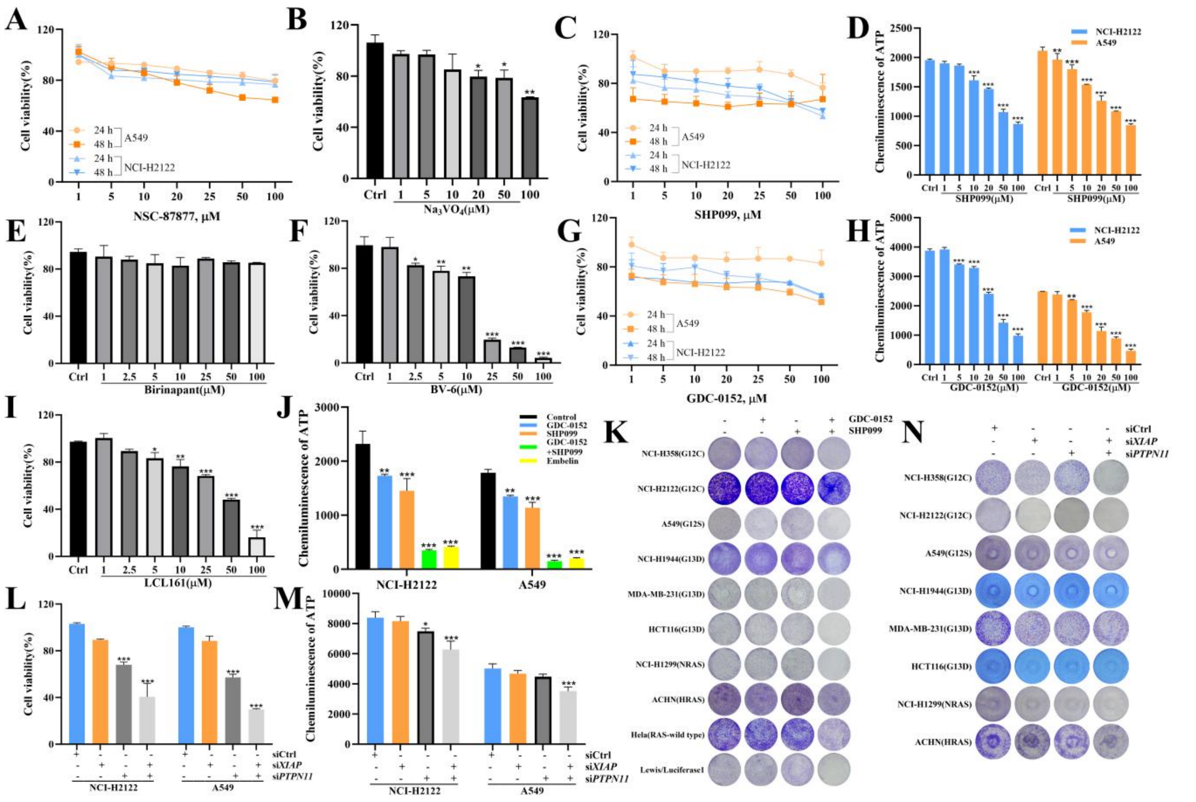


**Figure S7. Targeting SHP2 and XIAP produces a synthetic lethal effect.** (A) Effects of NSC-87877 on cell viability of A549 and NCI-H2122 cells after 24 and 48 h were determined using the CCK-8. Bars, ± SEM. The curves were plotted using a variable slope (four-parameter) non-linear fit. (B, E, F, and I) Effects of $Na_3VO_4$ (B), birinapant (E), BV-6 (F), and LCL161 (I) on cell viability of human NCI-H2122 cells at 48 h were determined using the CCK-8. $^{*}p < 0.05$, $^{**}p < 0.01$ and $^{***}p < 0.001$ compared to control group (unpaired two-tailed Student's *t*-test). (C, G) Effects of SHP099 (C) and GDC-0152 (G) on cell viability after 24 and 48 h were determined using the CCK-8. Bars, ± SEM. The curves were plotted using a variable slope (four-parameter) non-linear fit. (D, H) Effects of SHP099 (D) and GDC-0152 (H) on cell viability were determined after 48 h using intracellular ATP chemiluminescence. $^{**}p < 0.01$ and $^{***}p < 0.001$ compared to control group (unpaired two-tailed Student's *t*-test). (J) Effects of SHP099 and GDC-0152 separately and in combination, and the effect of embelin for 24 h on the cell viability were assessed using intracellular ATP chemiluminescence. $^{**}p < 0.01$ and $^{***}p < 0.001$ compared to control group (unpaired two-tailed Student's *t*-test). (K) Effects of SHP099 and GDC-0152 separately and in combination with SHP099 and SHP099 on the cell viability of human *KRAS*-WT and -mutant cells were assessed using a colony formation assay. (L, M) A549 and NCI-H2122 cells were instantaneously transfected with si*PTPN11* and si*XIAP* separately and together for 24 h under

3D cell culture (L) or single-layer cell mode (M), and intracellular ATP chemiluminescence or cell viability using the CCK-8 were tested. $^{***}p < 0.001$ compared to control or siCtrl group (unpaired two-tailed Student's *t*-test). (N) The human *KRAS*-WT and -mutant cells were transfected with si*PTPN11* and si*XIAP* separately and simultaneously for 72 h, and cell viability were detected using a colony formation assay. All experiments were conducted with three independent replicates.

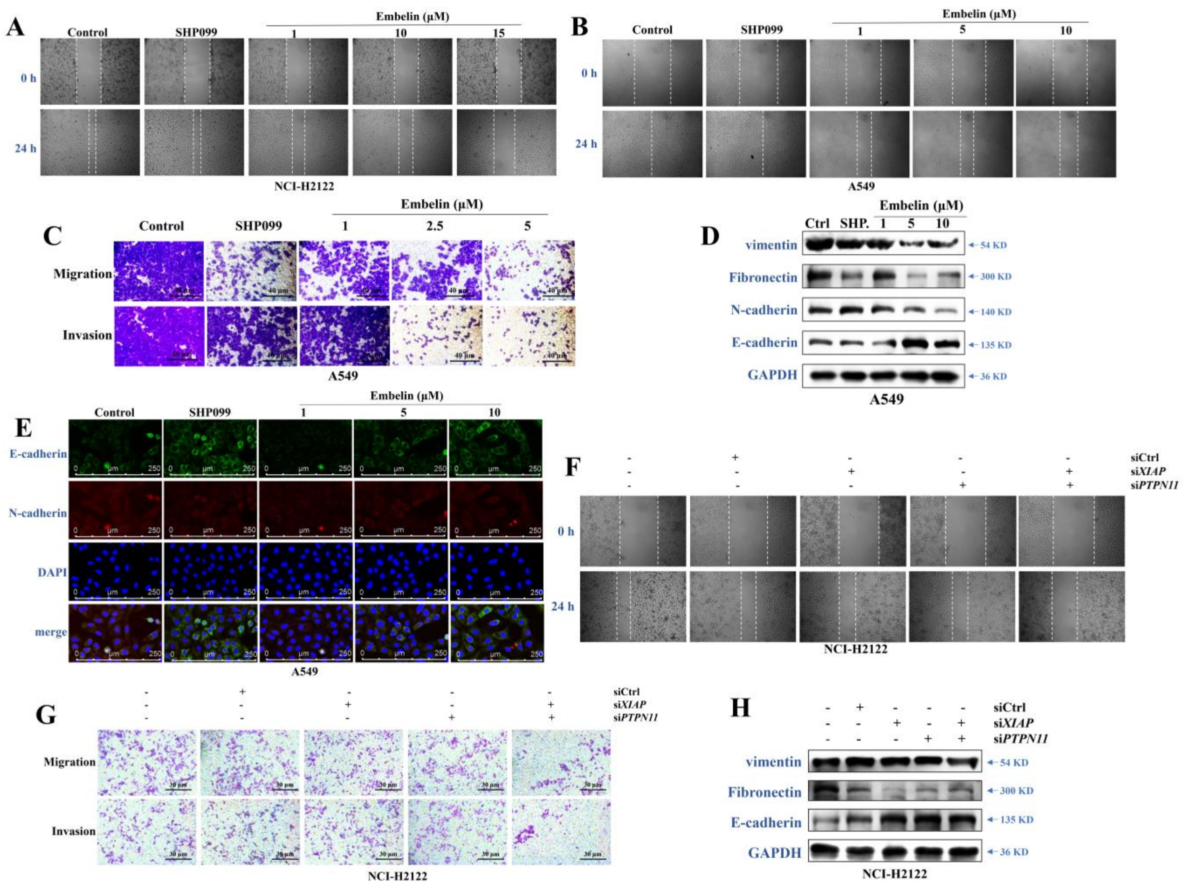


**Figure S8. Embelin inhibits cell migration and reverses EMT in *KRAS*-mutant NSCLC cells.** (A, B) Monolayers of NCI-H2122 (A) and A549 (B) cells were mechanically scratched and treated with embelin and SHP099 (10 μM) for 24 h, then the fusion distances were measured (×20). (C) A549 cells were inoculated into the upper lumen of the Transwell cell (upper layer of the cell was coated with Matrigel for invasion analysis) and treated with embelin and SHP099 (10 μM) for 24 h. Images of cell migration (above) and invasion (below) were captured using a microscope (×40). (D) Western blotting was used to assess protein expression of EMT markers, including E-cadherin, N-cadherin, vimentin, and fibronectin in A549 cells. (E) After treatment with embelin and SHP099 (10 μM) for 24 h, expression of EMT-labeled protein in A549 cells was measured using IF. (F) Effects of si*PTPN11* and si*XIAP* on scratch of NCI-H2122 cells after transient transfection for 24 h (×20). (G) Effects of si*PTPN11* and si*XIAP* on cell migration (above) and invasion (below) of NCI-H2122 cells after transient transfection for 24 h. (H) Effects of si*PTPN11* and si*XIAP* on EMT-related protein expression of NCI-H2122 cells after transient transfection for 24 h. All experiments were conducted with three independent replicates.

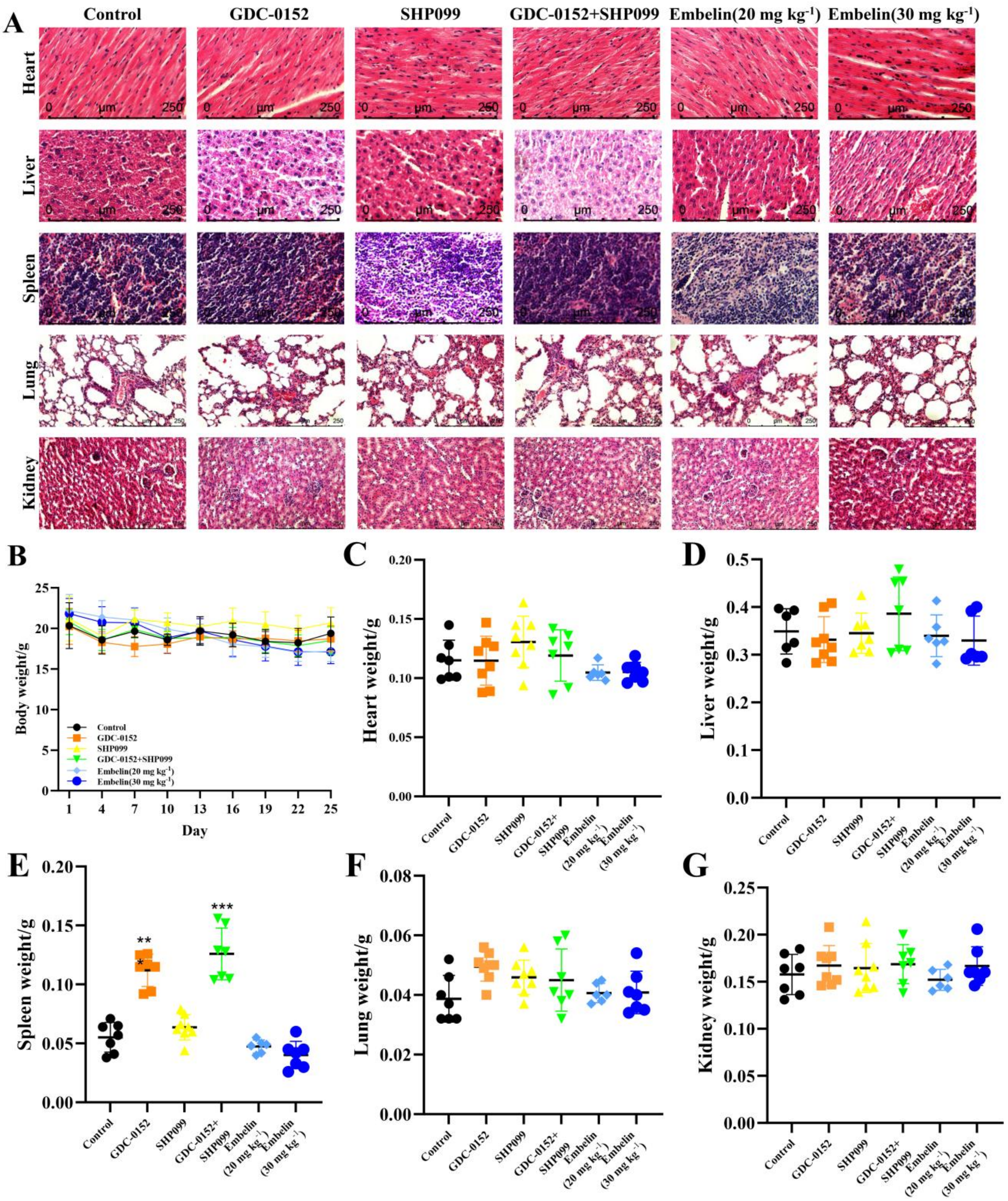


**Figure S9. Effects of embelin on morphological structure and weight of primary organs in NCI-H2122 subcutaneous tumor transplantation in BALb/c nude mice.** (A, C, D, E, F, and G) Major organs were excised from mice at the termination of the experiment. Representative images of H&E staining (A) of major organs (heart, liver, spleen, lungs, and kidneys were captured (×50 for heart, liver and spleen; ×100 for lung and kidney) and weights of the corresponding organs was measured (C, D, E, F, and G) in NCI-H2122 subcutaneous transplantation tumor BALb/c nude mice model (*n* = 6, each group).$^{**}p < 0.01$ compared to control and $^{***}p < 0.001$ compared to control group (unpaired two-tailed Student's *t*-test). (B)

Body weights of mice in the NCI-H2122 subcutaneous transplantation tumor BALb/c nude model were measured every 3 d. GraphPad was used to conduct statistical analysis of data (*n* = 6, each group).

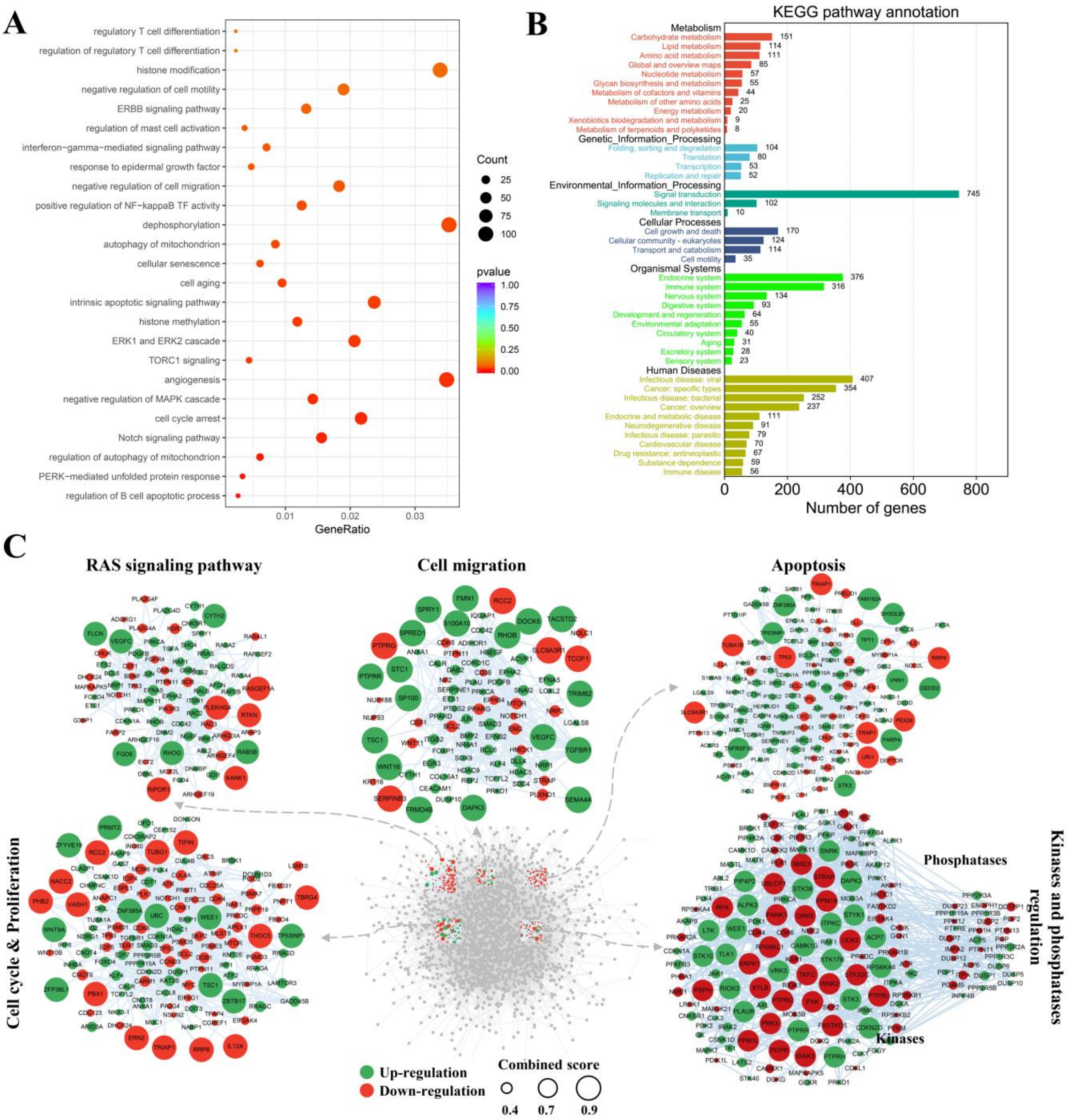


**Figure S10. Intracellular transcriptomic analysis of embelin on *KRAS*-mutant NCI-H2122 cells.** (A) Dot plot of the top 25 GO enrichment analysis between control and embelin-treated groups. (B) KEGG enrichment analysis involved in cellular processes, environmental information processing, genetic information processing, human diseases, metabolic processes, and organismal systems between the control and the embelin treatment groups. (C) Dot plot representation of the significantly enriched KEGG pathways of control and embelin-regulated phosphoproteins. Circle sizes represent the combined score of control and embelin-regulated phosphoproteins associated with the specific pathway and biological processes.

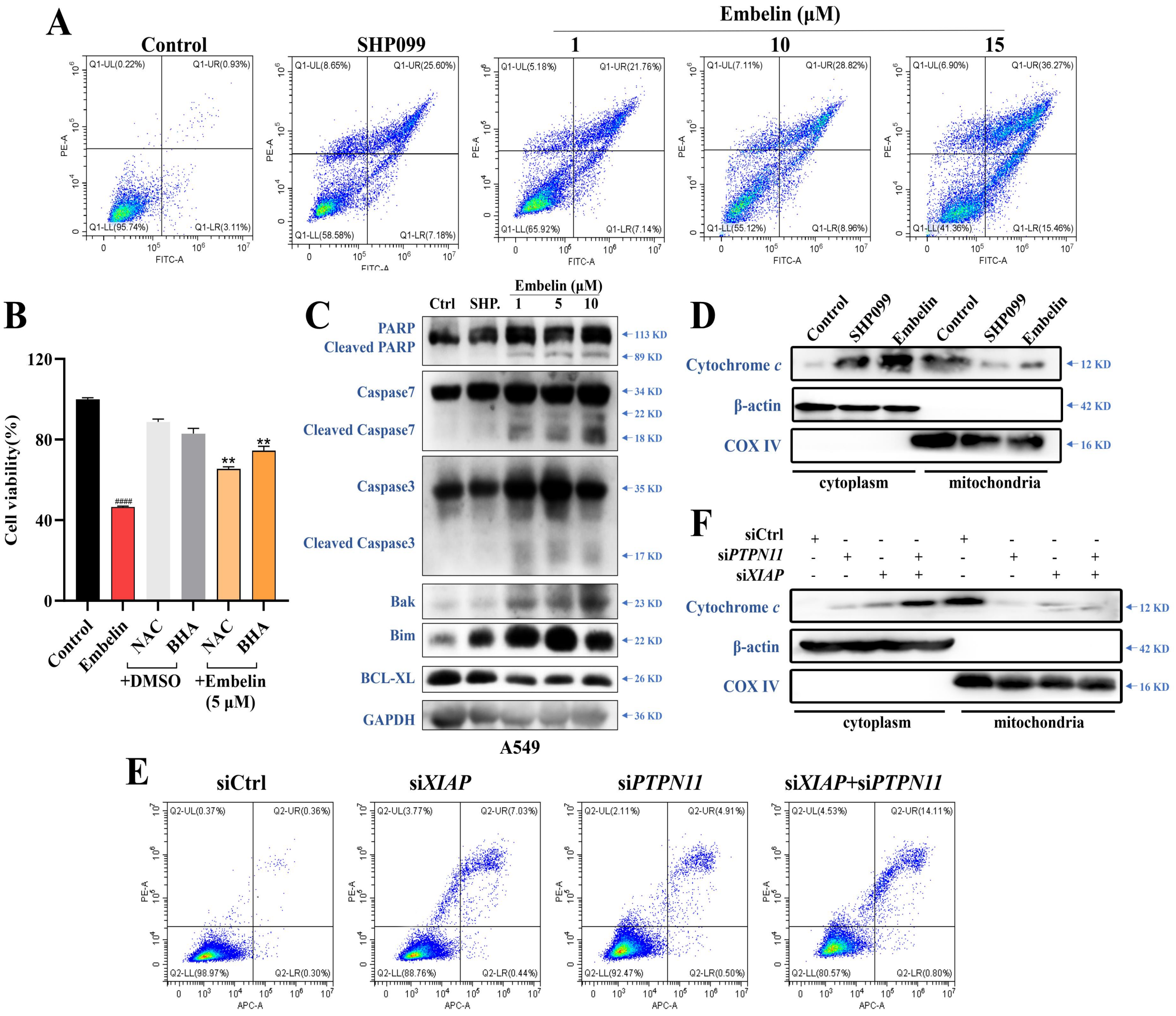


**Figure S11. Embelin induces cell apoptosis in *KRAS*-mutant NSCLC cells.** (A) NCI-H2122 cells were treated with embelin and SHP099 for 24 h and apoptotic cells were detected through Annexin-V-FITC/PI double staining. Representative flow cytometry plots are shown. (B) NCI-H2122 cells were treated with embelin and/or combination with ROS scavengers including n-acetylcysteine (NAC) and butylated hydroxyanisole (BHA) for 24 h, and cell viability was detected by CCK-8 assay. $^{\#\#\#\#}$ $p < 0.0001$ compared to control group; $^{**}$ $p < 0.01$ compared to embelin group (unpaired two-tailed Student's *t*-test). (C) A549 cells were treated with embelin and SHP099 (10 μM) for 24 h, and expressions of apoptosis-related proteins were measured using western blotting. (D) A549 cells were treated with embelin (15 μM) and SHP099 (10 μM) for 24 h and the levels of cytochrome *c* were measured using western blotting. (E) NCI-H2122 cells were instantaneously transfected with si*PTPN11* and si*XIAP* for 24 h, individual or both, and apoptotic cells were detected through Annexin-V-APC/PI double staining, with siCtrl as the control. Representative flow cytometry plots are shown. (F) A549 cells were instantaneously transfected with si*PTPN11* and si*XIAP* for 24 h, individual

or both, and cytochrome *c* release were assessed using western blotting, with siCtrl as the control. All experiments were conducted with three independent replicates.

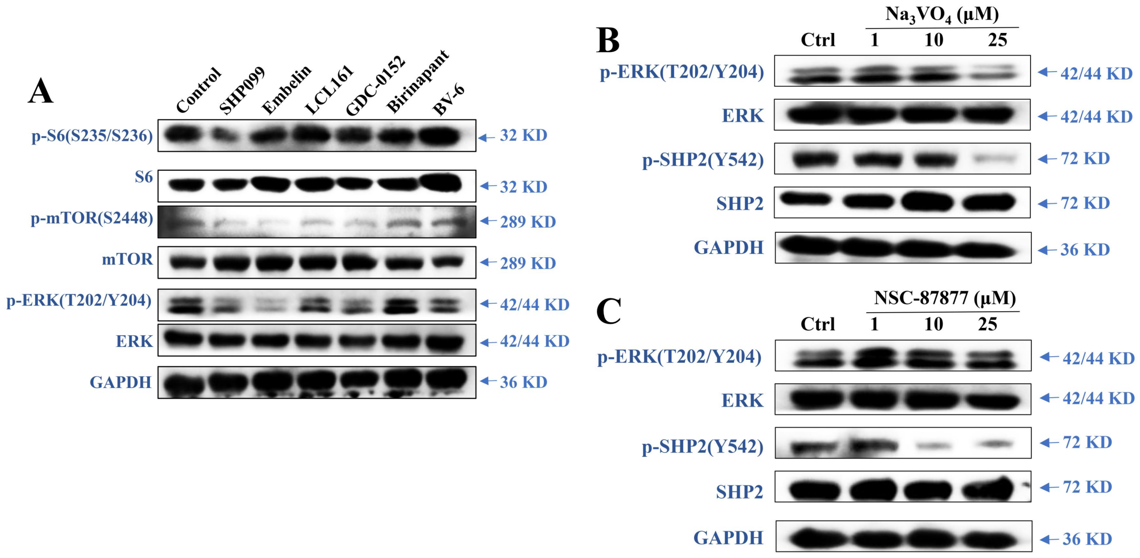


**Figure S12. Effects of SHP2 and XIAP inhibitors in signal pathway transduction.** (A) Effect of SHP099, embelin, and various XIAP inhibitors on expression and phosphorylation of ERK/MAPK and PI3K/AKT signaling pathways of NCI-H2122 cells were assessed using western blotting. (B, C) Protein expression and phosphorylation of RAS/MAPK signaling pathways in NCI-H2122 cells after treatment with $Na_3VO_4$ (B) and NSC-87877 (C) for 24 h were assessed using western blotting. All experiments were conducted with three independent replicates.

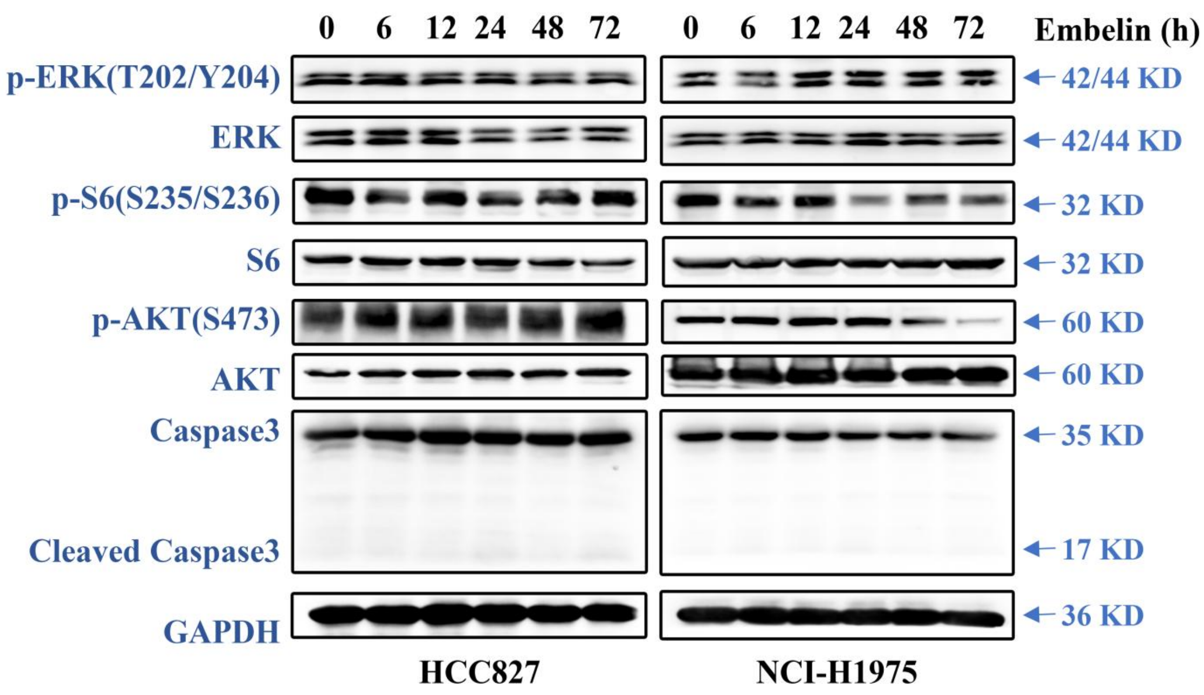


**Figure S13.** The effects of embelin at different time points on the expression and phosphorylation of ERK, S6, and AKT, as well as Caspase 3 activation in *KRAS* wild-type HCC827, NCI-H1975 cells. The experiments were conducted with three independent replicates.

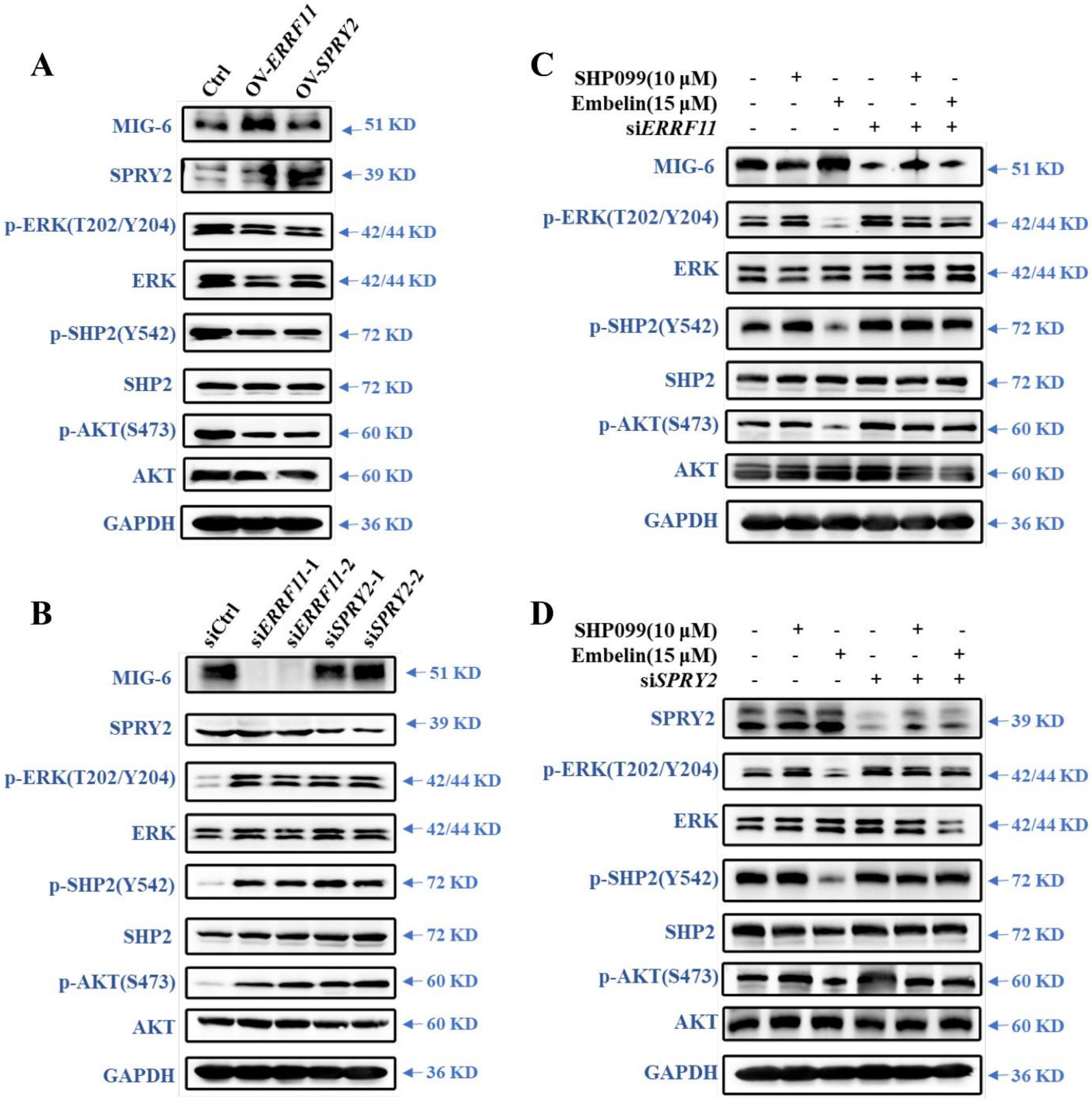


**Figure S14. MIG-6 and SPRY2 suppress the negative feedback effect.** (A, B) Overexpression (A) or knockdown (B) of MIG-6 and SPRY2 was performed in A549 cells, and the expression and phosphorylation levels of ERK, SHP2, and AKT proteins were analyzed by Western blotting. (C, D) MIG-6 (C) and SPRY2 (D) were knocked down in A549 cells after treating with embelin (15 µM) or SHP099 (10 µM) for 72 h, and western blotting was used to assess changes in the expression and phosphorylation levels of ERK, SHP2, and AKT. All experiments were conducted with three independent replicates.
