# Supplementary material for: Synthetic Lethality of SHP2 and XIAP Suppresses Proliferation and Metastasis in KRAS‐mutant Nonsmall Cell Lung Cancer
Source: Adv Sci (Weinh). 2025 Feb 24;12(15):2411642. doi: 10.1002/advs.202411642 (PMC12005787; doi:10.1002/advs.202411642)
Supplement: Supplementary file 2 — Supplementary Table1 [file ADVS-12-2411642-s002.pdf]

ADVANCED SCIENCE Open Access

Supporting Information

for *Adv. Sci.*, DOI 10.1002/advs.202411642

Synthetic Lethality of SHP2 and XIAP Suppresses Proliferation and Metastasis in *KRAS*-mutant Nonsmall Cell Lung Cancer

*Nai-jie Fu, Yu-wen Sheng, Zhe Fan, Zhao Wu, Ling-yu Li, Rui-ying Xi, Xiao-ke Shi, Guo-lin Zhang and Fei Wang**

Supplementary Table S1 Experimental related compounds and drugs

Reagents	Company Source
Preclinical/Clinical Compound Library	Selleck Chemicals, USA
SHP099	Selleck Chemicals, USA
$Na_3VO_4 \cdot 12H_2O$	Selleck Chemicals, USA
epidermal growth factor，EGF	Genscript, China
Embelin	Selleck Chemicals, USA
NSC-87877	Selleck Chemicals, USA
GDC-0152	Selleck Chemicals, USA
BV-6	Selleck Chemicals, USA
N-Acetyl-L-cysteine (NAC)	Sigma-Aldrich, USA
Tert butyl p-hydroxyanisole (BHA)	Yuanye, USA
Rotenone	Cayman Chemical, USA
Diphenyleneiodonium chloride (DPI)	Selleck Chemicals, USA
GKT-137831	Selleck Chemicals, USA
LCL-161	Selleck Chemicals, USA
TL32711	Selleck Chemicals, USA
Erlotinib	Selleck Chemicals, USA
Gefitinib	Selleck Chemicals, USA

Supplementary Table S2 Cell Experiment Related Reagents

Reagents	Company Source
si*PTPN11*	Tsingke Biotechnology, China
si*XIAP*	Tsingke Biotechnology, China
si*SPRY2*	Sangon Biotech, China
si*ERRF11*	Sangon Biotech, China
4 ',6-diamidino-2-phenylindole (DAPI) staining solution	Beyotime, China
Reactive oxygen detection kit	Beyotime, China
Mito-Tracker Red CMXRos Kit	Beyotime, China
Cell cycle and apoptosis detection kit	Beyotime, China
MitoSOX ™ Red mitochondrial superoxide kit	Thermo Fisher Scientific, USA
fetal bovine serum (FBS)	GIBCO，USA
RMPI-1640 medium	Biological Industries, Israel
DMEM medium	Hyclone, USA
Ham's F-12K medium	Sangon Biotech，China
Opti-MEM medium	GIBCO，USA
0.25% trypsin solution	Solarbio, China
phosphate buffered saline (PBS)	Solarbio, China
dimethyl sulfoxide (DMSO)	Sigma, USA
Cell counting kit-8 (CCK-8)	Beyotime, China
Cellular aging β-galactosidase staining kit	Beyotime, China

CellLight^{TM} Analysis Kit	Beyotime, China
penicillin/streptomycin (P/S)	Beyotime, China
crystal violet	Solarbio, China
TransIntro^{TM} EL transfection reagent	TransGen Biotech, China
Cytochrome *c* Release Assay Kit	Abcam, UK

Supplementary Table S3 Immunofluorescence Test Related Reagents

Reagents	Company Source
4% neutral formaldehyde solution	Sangon Biotech, China
Alexa Fluor 568 labeled goat anti rabbit IgG (H+L)	Beyotime, China
Alexa Fluor 555 labeled donkey anti mouse IgG (H+L)	Beyotime, China
Anti fluorescence quenching sealing agent	Beyotime, China
Tris-HCl（pH 7.4）	Solarbio, China
NP-40	Thermo Fisher Scientific, USA
Glycorol	Sangon Biotech, China
TBS（10×）	Beyotime, China
Protein A/G plus-agarose beads	Beyotime, China
Protein loading buffer (5 × Loading buffer)	Beyotime, China

Supplementary Table S4 Animal experiment related reagents

Reagents	Company Source
Iodophor	Runzekang Company, China
TUNEL Cell Apoptosis Detection Kit (Green Fluorescence)	Beyotime, China
4% cell tissue fixative	Sangon Biotech, China
Anhydrous ethanol	Kelong Chemical Factory, China
Xylene solution	Kelong Chemical Factory, China
Eosin Staining Solution	Solarbio, China
Hematoxylin staining Solution	Solarbio, China
Hydrogen peroxide (H_2O_2) solution	Kelong Chemical Factory, China
Sodium citrate powder	Zhongshan Jinqiao Co., Ltd, China
Neutral resin sealing agent	Solarbio, China
Liquid Blocker Super PAP Pen	Beyotime, China
Anti Mouse/Rabbit Immunohistochemical Detection Kit	Proteintech, China
Protease K solution	Solarbio, China
Anti-Ki 67 Mouse Monoclonal Antibody	SAB, USA
Tween-80	Solarbio, China
PEG300	MCE, USA

Supplementary Table S5 Western Blotting Experiment Related Reagents

Reagents	Company Source
radio immunoprecipitation assay (RIPA) lysis solution	Beyotime, China
30% acrylamide solution	Sangon Biotech, China
Ammonium persulfate (APS)	Sangon Biotech, China
Sodium dodecyl sulfate (SDS)	Sangon Biotech, China
tetramethylethylenediamine (TEMED)	Sangon Biotech, China
Tris base	Sangon Biotech, China
hydrochloric acid (HCl)	Kelong Chemical Factory, China
Glycin	Sangon Biotech, China
Tween-20	Solarbio, China
methanol	Kelong Chemical Factory, China
Blue Plus® II Protein Marker (14-120 kDa)	TransGen Biotech, China
Hypersensitive ECL Chemiluminescence Substrate	4A Biotech Co., Ltd, China
Nitrocellulose (NC) membrane	Millipore, China
Labeling goat anti mouse IgG with horseradish peroxidase	Zhongshan Jinqiao Co., Ltd, China
Labeling goat anti rabbit IgG with horseradish peroxidase	Zhongshan Jinqiao Co., Ltd, China
Anti-XIAP antibody	Proteintech, China
Anti-p-Gab1(Y660) antibody	SAB, USA
Anti-STAT3 antibody	Huaan, China
Anti-p-STAT3(S727) antibody	SAB, USA
Anti-P38 antibody	Cell Signaling Technology, USA
Anti-p-P38 (T180/Y182) antibody	Cell Signaling Technology, USA
Anti-JNK antibody	Proteintech, China
Anti-p-JNK(T183/Y185) antibody	Sangon Biotech, China
Anti-PARP antibody	Proteintech, China
Anti-Cleaved PAR*P* antibody	Proteintech, China
Anti-Caspase3 antibody	Cell Signaling Technology, USA
Anti-Bid antibody	Cell Signaling Technology, USA
Anti-Bak antibody	Cell Signaling Technology, USA
Anti-Bcl-XL antibody	Cell Signaling Technology, USA
Anti-SHP2 antibody	Proteintech, China
Anti-p-SHP2(Y542) antibody	Proteintech, China
Anti-p-SHP2(Y580) antibody	Proteintech, China
Anti-MEK1/2 antibody	Proteintech, China
Anti-ERK1/2 antibody	Proteintech, China
Anti-p-ERK1/2(T202/Y204) antibody	Proteintech, China
Anti-AKT antibody	Proteintech, China
Anti-p-AKT(S473) antibody	Proteintech, China
Anti-E-cadherin antibody	Proteintech, China
Anti-N-cadherin antibody	Proteintech, China

Anti-Fibronectin antibody	Proteintech, China
Anti-MIG-6 antibody	Proteintech, China
Anti-SPRY2 antibody	Proteintech, China
Anti-Gab1 antibody	Proteintech, China
Anti-Cleaved Caspase 3 antibody	Proteintech, China
Anti-Cleaved Caspase 7 antibody	Proteintech, China
Anti-vimentin antibody	Proteintech, China
Anti-S6 antibody	SAB, USA
Anti-p-MEK1/2(S217/221) antibody	SAB, USA
Anti-Grb2 antibody	SAB, USA
Anti-mTOR antibody	SAB, USA
Anti-GAPDH antibody	Bioworld Technology, China
Anti-SOS1 antibody	Abclonal, China
Anti-p-S6(S235/236) antibody	Abclonal, China
Anti-p-mTOR(S2448) antibody	Affinity Bioscience, China
Anti-EGFR antibody	Proteintech, China
Anti-p-EGFR(Y1068) antibody	Bioworld technology, USA
Anti-β-catenin antibody	Proteintech, China
Anti-p-β-catenin(T41) antibody	SAB, USA
Anti-NF-κB p65 antibody	SAB, USA
Anti-p-NF-κB p65(S536) antibody	SAB, USA
Anti-β-actin antibody	Proteintech, China
Anti-COX IV antibody	SAB, USA

Supplementary Table S6 Protein Purification Experiment Related Reagents

Reagents	Company Source
chloramphenicol (Chl)	Sangon Biotech, China
SHP2 activated peptide	Sangon Biotech, China
DTT Substitutes（TCEP）	Beyotime, China
Agarose	Solarbio, China
Ampicillin (Amp)	Sangon Biotech, China
Kanamycin (Kana)	Sangon Biotech, China
Trans5 α Chemoreceptive cell	TransGen Biotech, China
BL21 Chemosensory Cells	TransGen Biotech, China
Isopropyl-β-D-thiogalactose（IPTG）	Biofroxx, Germany
SHP2 cDNA	Sino Biological, China
Protein tyrosine phosphatase 1 containing SH-2 domain (SHP1) cDNA	Sino Biological, China
Protein tyrosine phosphatase 2 containing SH-2 domain (SHP2) cDNA	Sino Biological, China

Protein tyrosine phosphatase 1B (PTP1B) cDNA	Sino Biological, China
T cell protein tyrosine phosphatase (TCPTP) cDNA	Sino Biological, China
Human pox vaccine H1 related phosphatase (VHR) cDNA	Sino Biological, China
Hematopoietic protein tyrosine phosphatase (HePTP) cDNA	Sino Biological, China
PGEX4T1 plasmid	Novagen, Britain
pET28a plasmid	Novagen, Britain
pET32a plasmid	Novagen, Britain
6,8-difluoro-4-methyl umbelliferyl phosphate (DiFMUP)	Invitrogen, USA
BCA detection and analysis kit	Beyotime, China
yeast extract	Oxoid, Britain
tryptone	Oxoid, Britain
lysozyme	Beyotime, China
phenylmethanesulfonyl fluoride (PMSF)	Beyotime, China
4 - (2-hydroxyethyl) - 1-piperazine ethanesulfonic acid (HEPES)	Sigma-Aldrich, USA
Ethylenediamine tetraacetic acid (EDTA)	Sigma-Aldrich, USA
Dithiothreitol (DTT)	Sigma-Aldrich, USA
Triton X-100	Solarbio, China
bovine serum albumin (BSA)	Solarbio, China
glutathione (GSH)	Beyotime, China
NaCl	Beyotime, China
imidazole	Solarbio, China
Coomassie Brilliant Blue R-250	Solarbio, China
DNA FastDigest Enzyme	Thermo Fisher Scientific, USA
Supercoiled DNA Ladder Marker	Thermo Fisher Scientific, USA
DNA marker	TransGen Biotech, China
Ni-Agarose Resin	Cowin Bio, China
BeyoGold™ GST-tag Purification Resin	Beyotime, China

Supplementary Table S7 Inhibitory effects of Embelin on *KRAS*-mutant and wild-type cells. (Mean ± SD)

Cell Lines	Cell type	Mutant type	Mutant site	IC_{50} at 24 h (μM)	IC_{50} at 48 h (μM)
NCI-H2122	Lung cancer	KRAS	G12C	4.9 ± 0.1	4.03 ± 0.1
NCI-H1944	Lung cancer	KRAS	G13D	31.0 ± 0.2	21.6 ± 0.9
NCI-H358	Lung cancer	KRAS	G12C	12.2 ± 0.4	9.2 ± 0.3
NCI-H1299	Lung cancer	NRAS	-	19.6 ± 1.0	13.1 ± 0.6
A549	Lung cancer	KRAS	G12S	7.9 ± 0.8	6.5 ± 0.9
MDA-MB-231	Breast cancer	KRAS	G13D	13.6 ± 0.2	11.5 ± 0.7

HCT116	Colon cancer	KRAS	G13D	18.2 ± 0.9	9.6 ± 0.4
ACHN	Renal cancer	HRAS	-	27.4 ± 0.3	10.3 ± 0.7
HeLa	Cervical cancer	RAS-wild type	-	16.5 ± 0.2	13.4 ± 0.6
Lewis/Luc1	Lung cancer	RAS-wild type	-	20.6 ± 0.6	18.2 ± 0.9
NCI-H1693	Lung cancer	RAS-wild type	-	42.57 ± 0.34	16.16 ± 0.37
NCI-H838	Lung cancer	RAS-wild type	-	39.79 ± 0.27	10.54 ± 0.44
NCI-H1915	Lung cancer	HRAS	-	60.6 ± 0.41	11.41 ± 0.39
NCI-H1755	Lung cancer	RAS-wild type	-	49.4 ± 0.32	14.23 ± 0.27
NCI-H1155	Lung cancer	KRAS	Q61H	16.14 ± 0.36	7.07 ± 0.45
SNU-C2A	Colorectal cancer	KRAS	G12D	22.37 ± 0.5	8.73 ± 0.43
HCT15	Colorectal cancer	KRAS	G13D	12.85 ± 0.43	9.95 ± 0.38
HCC44	Lung cancer	KRAS	G12C	27.28 ± 0.44	6.52 ± 0.4
SW620	Colorectal cancer	KRAS	G12V	18.61 ± 0.4	5.06 ± 0.45
SW1116	Colorectal cancer	KRAS	G12A	19.48 ± 0.29	7 ± 0.35
SW1417	Colorectal cancer	RAS-wild type	-	65.39 ± 0.36	14.93 ± 0.34
NCI-H1975	Lung cancer	RAS-wild type	-	7.40 ± 0.83	-
HCC827	Lung cancer	RAS-wild type	-	4.95 ± 0.28	-
